# Redox‐Induced Stabilization of AMBRA1 by USP7 Promotes Intestinal Oxidative Stress and Colitis Through Antagonizing DUB3‐Mediated NRF2 Deubiquitination

**DOI:** 10.1002/advs.202411320

**Published:** 2025-01-31

**Authors:** Weimin Xu, Zhebin Hua, Yaosheng Wang, Wenbo Tang, Wensong Ge, YingWei Chen, Zhongchuan Wang, Yubei Gu, Chen‐Ying Liu, Peng Du

**Affiliations:** ^1^ Department of Colorectal Surgery Xinhua Hospital Shanghai Jiaotong University School of Medicine Shanghai 200092 China; ^2^ Shanghai Colorectal Cancer Research Center Shanghai 200092 China; ^3^ Department of Gastroenterology Xinhua Hospital Shanghai Jiaotong University School of Medicine Shanghai 200092 China; ^4^ Department of Gastroenterology Rui Jin Hospital Affiliate to Shanghai Jiao Tong University school of Medicine 197 Rui Jin Er Road Shanghai 200025 China

**Keywords:** AMBRA1, inflammatory bowel disease, intestinal oxidative stress, NRF2, USP7

## Abstract

Inflammatory bowel disease (IBD) is associated with oxidative stress and redox signaling disruption. It is recently reported that proautophagic autophagy/beclin‐1 regulator 1 (AMBRA1) is a positive modulator of the NF‐κB pathway that promotes intestinal inflammation. However, its effect on intestinal redox state and whether AMBRA1 is regulated by oxidative stress remain unknown. In this study, it is found that AMBRA1 functions as a pro‐oxidative factor that increases oxidative stress in intestinal epithelial cells (IECs) in vitro and in vivo. Mechanistically, the N‐terminal F1 domain is required for AMBRA1 to competitively interact with the N‐terminal domain of NRF2, thereby antagonizing the interaction between deubiquitinating protein 3 (DUB3) and NRF2, suppressing DUB3‐mediated NRF2 deubiquitination, and leading to NRF2 degradation. In response to H_2_O_2_ stimulation, the interaction between AMBRA1 and ubiquitin‐specific protease 7 (USP7) is enhanced, facilitating USP7 to deubiquitinate AMBRA1 at K83 and K86 and stabilize AMBRA1. Notably, the USP7 inhibitor, P5091, inhibits oxidative stress and colitis in vivo. Elevated AMBRA1 expression in inflamed colon tissues from ulcerative colitis patients is negatively correlated with decreased NRF2 protein levels. Overall, this study identifies AMBRA1 as a pro‐oxidative factor in IECs and provides a redox‐modulating therapeutic strategy for targeting USP7/AMBRA1 in IBD.

## Graphical abstract

This study not only reveals a new KEAP1‐independent mechanism of NRF2 protein stability via disruption of NRF2/DUB3 complex by AMBRA1 in intestinal epithelial cells, but also highlights USP7 as a deubiquitinase of AMBRA1 in response to oxidative stress and the therapeutic potential of targeting USP7‐AMBRA1 axis for inflammatory bowel disease treatment.

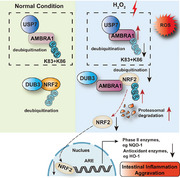

## 1 Introduction

Inflammatory bowel disease (IBD), which includes Crohn's disease (CD) and ulcerative colitis (UC), is a chronic nonspecific intestinal inflammatory disease with a chronic course of recurrent relapse and remission.^[^ ^1^, ^2^, ^3^ ^]^ Although biological therapies, such as antitumor necrosis factor‐α (TNF‐α) therapy, have been widely used and have shown satisfactory efficacy in clinical settings, a significant number of patients do not benefit from these therapies or fail to maintain long‐term mucosal healing and clinical remission. This highlights the complexity of the etiology and pathology of IBD and the urgent need to develop novel therapies for IBD.^[^ ^4^, ^5^, ^6^ ^]^ IBD arises as a result of complicated interactions between genetic and environmental factors that lead to dysregulated immune responses and mucosal inflammatory damage in the intestine.^[^ ^3^, ^7^ ^]^ The inflamed mucosa is typically infiltrated by numerous inflammatory cells, such as activated macrophages and neutrophils, leading to sustained overproduction and release of reactive oxygen species (ROS), subsequent oxidative damage to intestinal tissues and an enhanced inflammatory response.^[^ ^8^, ^9^, ^10^ ^]^ Thus, IBD is accompanied by oxidative stress and the dysregulation of redox signaling^[^ ^8^, ^9^, ^10^ ^]^ Antioxidants can be used as adjunct therapies in IBD.^[^ ^10^ ^]^

The Kelch‐like ECH‐associated protein 1 (KEAP1)/nuclear factor erythroid 2‐related factor 2 (NRF2) pathway is a vital antioxidant signaling pathway that preserves cellular homeostasis and is a promising therapeutic target for IBD.^[^ ^8^, ^9^, ^11^ ^]^ The transcription factor NRF2 is primarily modulated by the KEAP1‐CUL3 E3 ligase complex, which promotes the Lys48‐linked polyubiquitination of NRF2 and its subsequent proteasome‐dependent degradation.^[^ ^9^, ^11^ ^]^ Oxidative stress releases NRF2 from KEAP1 and induces its nuclear accumulation, where it activates the transcription of many antioxidant response element (ARE)‐dependent genes, including the antioxidant enzymes heme oxygenase‐1 (HO‐1) and quinone oxidoreductase 1 (NQO1).^[^ ^12^ ^]^ Thus, the activation of the KEAP1/NRF2 pathway protects intestinal epithelial cells (IECs) from oxidative damage, and accumulating evidence has shown that the activation of NRF2 can relieve intestinal inflammation, restore intestinal barrier integrity, and augment antioxidant defense. In addition to the KEAP1‐CUL3 E3 complex, NRF2 protein stability is modulated by the β‐transducin repeat‐containing protein (β‐TrCP) E3 complex.^[^ ^13^ ^]^ Deubiquitination also regulates NRF2 stability. Both ubiquitin‐specific protease 15 (USP15) and USP7 suppress NRF2 activity through the deubiquitination of KEAP1.^[^ ^14^, ^15^ ^]^ Inhibition of USP15 or USP7 leads to the activation of NRF2, resulting in impaired leukemic cell function in acute myeloid leukemia and attenuated neuroinflammation in neurodegenerative disease.^[^ ^15^, ^16^ ^]^ Deubiquitinating protein 3 (DUB3) was recently identified as a deubiquitinase that directly modulates NRF2 ubiquitination and subsequent degradation.^[^ ^17^ ^]^ However, the underlying mechanism through which the DUB3‐NRF2 regulatory axis exerts its effects and the related pathological and translational implications in IBD remain obscure.

Autophagy plays a vital role in the oxidative stress response and participates in crosstalk with the KEAP1/NRF2 pathway.^[^ ^18^, ^19^ ^]^ One NRF2 downstream target, p62/SQSTM1, binds competitively to KEAP1 and promotes autophagy‐dependent degradation of KEAP1 and activation of NRF2.^[^ ^19^ ^]^ Autophagy/beclin‐1 regulator 1 (AMBRA1) is a pro‐autophagic protein that initiates autophagy by promoting the ubiquitination and activity of ULK1 and BECN1.^[^ ^20^, ^21^ ^]^ As a highly intrinsically disordered adapter protein conserved in vertebrates, AMBRA1 has diverse and important autophagy‐independent functions through its interactions with multiple proteins**.^[^** ^20^ ^]^ A genome‐wide association study reported that the rs11819869 SNP in AMBRA1 was associated with inflammatory manifestations in patients with Crohn's disease (CD),^[^ ^22^ ^]^ suggesting a potential function of AMBRA1 in IBD. We recently demonstrated that AMBRA1 promoted intestinal inflammation in an autophagy‐independent manner by inhibiting PP4R1/PP4c‐mediated IKK dephosphorylation.^[^ ^23^ ^]^ However, the relationship between biological functions of AMBRA1 and intestinal oxidative stress remains largely unknown.

In the present study, we report that AMBRA1 is a negative regulator of the antioxidant response transcription factor NRF2 in IECs. AMBRA1 promotes NRF2 degradation by competitively antagonizing the interaction between NRF2 and DUB3, thereby inhibiting DUB3‐mediated deubiquitination and stabilizing NRF2. AMBRA1 protein expression is upregulated in inflamed colon tissues and negatively correlated with NRF2, HO‐1, and NQO‐1 levels. H_2_O_2_ stimulation enhances the interaction between USP7 and AMBRA1 and facilitates deubiquitination of AMBRA1 by USP7, leading to increased AMBRA1 protein abundance in response to oxidative stress. Importantly, the specific UPS7 inhibitor P5091 effectively inhibits intestinal oxidative stress and alleviates dextran sulfate sodium (DSS)‐induced colitis in mice. Our study revealed the suppressive function of AMBRA1 in the NRF2‐mediated antioxidant response and the dysregulated mechanism of AMBRA1 deubiquitination in the oxidative stress response, highlighting the therapeutic potential of the targeted inhibition of AMBRA1 or USP7 for the treatment of IBD.

## 2 Results

### 2.1 AMBRA1 Exacerbates H_2_O_2_‐Induced Oxidative Stress in IECs

**Figure 1:**
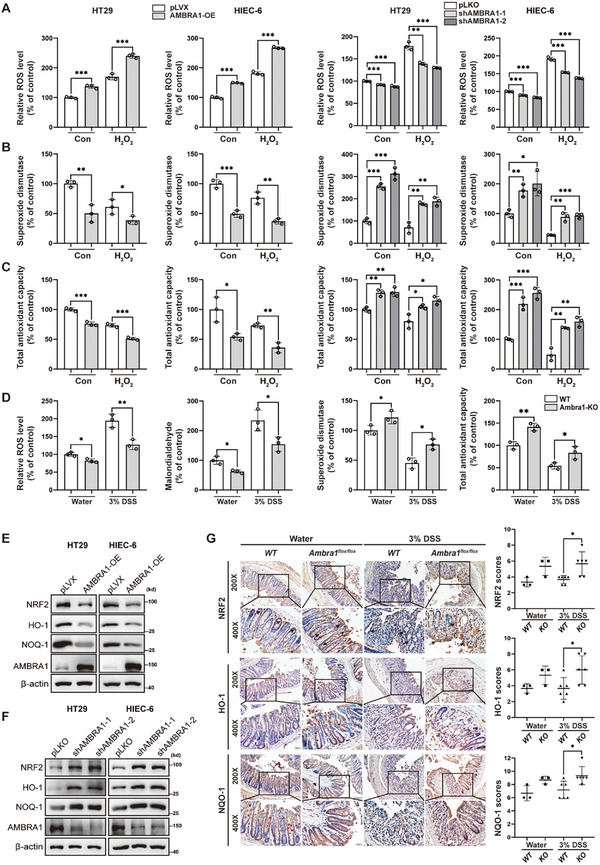
AMBRA1 enhances intestinal oxidative stress in vitro and in vivo. A–C) The levels of ROS (A), SOD (B) and T‐AOC (C) were measured in AMBRA1‐overexpressing, AMBRA1‐deleted and control HT29, HIEC‐6 cells after treatment with H_2_O_2_ (200 µM) for 6 h by using commercially available kits according to the manufacturer's instructions. D) The levels of ROS, MDA, SOD and T‐AOC were measured in intestinal epithelium from in control and DSS‐treated *WT* and *Villin‐Ambra1^flox/flox^* mice by using commercially available kits according to the manufacturer's instructions. DSS‐induced *WT* and *Villin‐Ambra1^flox/flox^* mice were littermate. E,F) Western blot analysis of NRF2 expression in AMBRA1‐overexpressing, AMBRA1‐knockdown and control HT29 and HIEC‐6 cells. G) NRF2, HO‐1 and NQO‐1 expression were examined, evaluated and semiquantitatively scored in colons from control and DSS‐treated *WT* and *Villin‐Ambra1^flox/flox^* mice via immunohistochemistry. The data are presented as the means ± SDs. n = 3 (A–D) biologically independent samples per group. These data (A–C) are representative of 3 independent experiments. n = 3 biologically independent samples for control group, n = 6 biologically independent samples for DSS group G). One‐way ANOVA (A–D) and the Mann‒Whitney's U test (G) were performed to assess statistical significance. * *P* < 0.05, ** *P* < 0.01, *** *P* < 0.001.

To determine the biological function of AMBRA1 in intestinal oxidative stress, we established stable HT29 and HIEC‐6 cells with AMBRA1 overexpression or knockdown (Figure S1A,B, Supporting Information) and measured the levels of ROS, superoxide dismutase (SOD) activity, and total antioxidant capacity (T‐AOC) upon H_2_O_2_ treatment. H_2_O_2_ treatment increased ROS levels but decreased SOD and T‐AOC levels in control HT29 and HIEC‐6 cells (**Figure** **1**A–C). Strikingly, AMBRA1 overexpression further increased ROS levels, whereas AMBRA1 knockdown decreased ROS levels in HT29 and HIEC‐6 cells after H_2_O_2_ treatment (Figure 1A). Consistent with the above observations, the levels of SOD and T‐AOC further decreased in HT29 and HIEC‐6 cells overexpressing AMBRA1 (Figure 1B,C). In contrast, the decrease in SOD and T‐AOC levels after H_2_O_2_ treatment was attenuated by the AMBRA1 knockdown (Figure 1B,C). Notably, similar results were observed in HT29 and HIEC‐6 cells under normal culture conditions, suggesting that AMBRA1 alone induces oxidative stress in IECs (Figure 1A–C). Our recent study demonstrated that AMBRA1 knockout in IECs attenuated DSS‐induced acute colitis.^[^ ^23^ ^]^ Therefore, we further explored the effects of AMBRA1 on intestinal oxidative stress during colitis in vivo. By examining oxidative and antioxidant markers, we found that compared with those from *WT* littermates, intestinal tissues from *Villin‐Ambra1^flox/flox^* mice presented lower levels of ROS and MDA and higher levels of SOD and T‐AOC after 7 days of DSS induction (Figure 1D).

Next, we examined the effects of AMBRA1 on NRF2 and related cellular antioxidant responses. Intriguingly, we observed that AMBRA1 overexpression drastically decreased the levels of NRF2, HO‐1, and NQO‐1 proteins in HT29 and HIEC‐6 cells (Figure 1E). In contrast, the levels of these proteins increased in both AMBRA1‐knockdown HT29 and HIEC‐6 cells (Figure 1F). Immunohistochemical analysis confirmed that the expression of NRF2, HO‐1, and NQO‐1 in the intestine was markedly greater in *Villin‐Ambra1^flox/flox^* mice than in their *WT* littermates after DSS induction (Figure 1G). Collectively, these results indicated that AMBRA1 exacerbates oxidative stress during intestinal inflammation, likely through the suppression of NRF2 expression.

### 2.2 AMBRA1 Promotes NRF2 Degradation in a KEAP1‐Independent Manner

**Figure 2:**
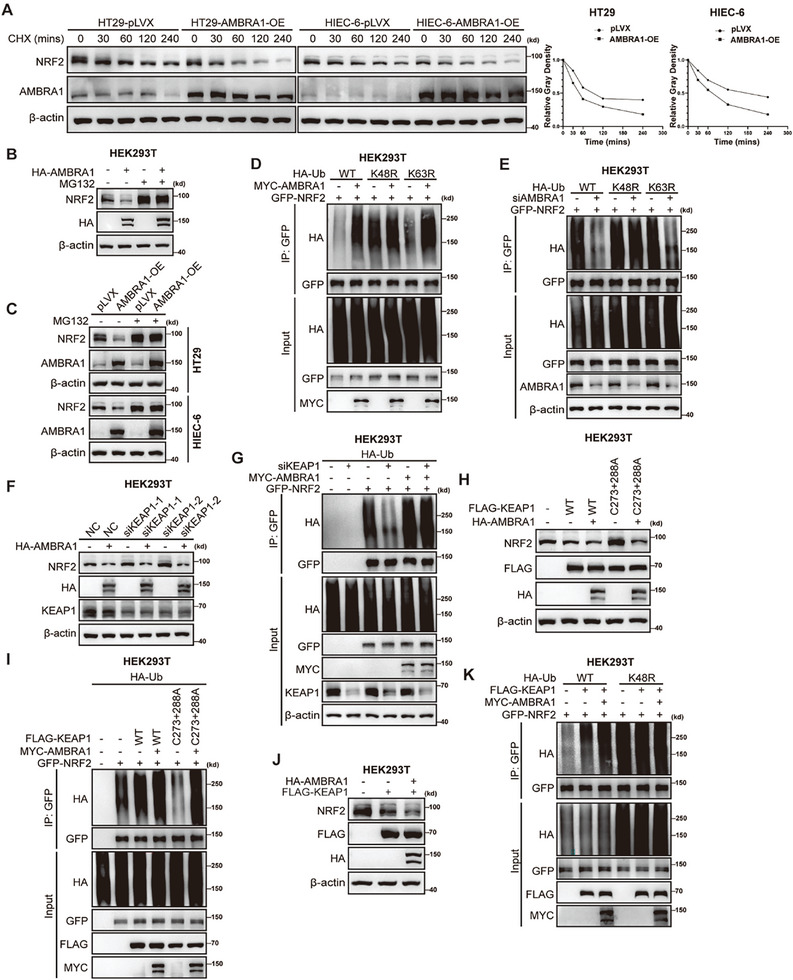
AMBRA1 promotes NRF2 degradation via K48‐linked polyubiquitination in a KEAP1‐independent manner. A) Analysis of the half‐life of NRF2 protein in control and AMBRA1‐overexpressing HT29 and HIEC‐6 cells. The cells were treated with cycloheximide (CHX) (75 µg ml^−1^) for the indicated times before western blot analysis of NRF2 protein levels. B) The protein level of NRF2 was examined by western blotting in HEK293T cells transfected with indicated plasmids and treated with or without MG132 (10 µM) treatment. To enhance the detection of NRF2 protein in this study, 60–80 µg total protein were used for western blot analysis of the NRF2 protein in HEK293T cells. C) The protein levels of NRF2 were measured by western blotting in control and AMBRA1‐overexpressing HT29 and HIEC‐6 cells with or without MG132 (10 µM) treatment. D) HEK293T cells were transfected with HA‐Ub, HA‐Ub‐K48R, HA‐Ub‐K63R, GFP‐NRF2 and MYC‐AMBRA1. GFP‐NRF2 was immunoprecipitated for subsequent detection and quantification of the ubiquitination level via western blotting. E) The control and AMBRA1‐knockdown HEK293T cells were transfected with HA‐Ub, HA‐Ub‐K48R, HA‐Ub‐K63R and GFP‐NRF2. GFP‐NRF2 was immunoprecipitated for subsequent quantification of the ubiquitination level of NRF2 via western blotting. F) HEK293T cells were transfected with control and siRNA targeting KEAP1 and then transfected with the indicated plasmids. Two days after transfection, NRF2 expression was determined via western blotting. G) The control and KEAP1‐knockdown HEK293T cells were transfected with HA‐Ub, MYC‐AMBRA1 and GFP‐NRF2 plasmids. Lysates of the cells were used for immunoprecipitation with an anti‐GFP antibody for subsequent quantification of NRF2 ubiquitination via western blotting. H) HEK293T cells were transfected with the indicated control vector, FLAG‐KEAP1^WT^, FLAG‐KEAP1^C273&C288A^ and HA‐AMBRA1 plasmids. NRF2 protein levels were measured via western blotting. I) HEK293T cells were transfected with HA‐Ub, FLAG‐KEAP1^WT^, FLAG‐KEAP1^C273&C288A^, MYC‐AMBRA1 and GFP‐NRF2 plasmids. The cell lysates were used for immunoprecipitation with an anti‐GFP antibody for subsequent quantification of NRF2 ubiquitination via western blotting. J) HEK293T cells were transfected with the indicated control vector or with the FLAG‐KEAP1 or HA‐AMBRA1 plasmid. NRF2 protein levels were measured via western blotting. K) HEK293T cells were transfected with the indicated HA‐Ub, HA‐Ub‐K48R, GFP‐NRF2, MYC‐AMBRA1 and FLAG‐KEAP1 plasmids. The level of NRF2 ubiquitination was analyzed as described above.

To clarify the mechanism through which AMBRA1 downregulates NRF2 expression, we measured NRF2 mRNA levels and protein half‐life in AMBRA1‐overexpressing cells. The NRF2 mRNA levels were not altered by the overexpression or knockdown of AMBRA1 (Figure S2A, Supporting Information). Under basal conditions, NRF2 is an unstable protein with a half‐life ranging from approximately 20 to 120 min in different cell lines, depending on the cellular context.^[^ ^24^, ^25^, ^26^ ^]^ The half‐lives of NRF2 in HT29 and HIEC‐6 cells were 70 and 120 min, respectively. Furthermore, a cycloheximide (CHX) assay revealed that AMBRA1 overexpression moderately decreased the half‐life of endogenous NRF2 in both HT29 and HIEC‐6 cells (**Figure** **2**A). In addition, the decrease in NRF2 protein levels caused by AMBRA1 overexpression was abrogated by the proteasome inhibitor MG132 in HEK293T, HT29, and HIEC‐6 cells, suggesting that AMBRA1 promotes proteasome‐mediated protein degradation of NRF2 (Figure 2B,C). As expected, the ubiquitination assay results revealed that AMBRA1 markedly promoted K48‐linked, but not K63‐linked, ubiquitination of NRF2 (Figure 2D). AMBRA1 knockdown decreased the K48‐linked ubiquitination of NRF2 (Figure 2E).

KEAP1‐CUL3 ligase complex targets NRF2 for ubiquitin‐dependent proteasomal degradation.^[^ ^9^ ^]^ Thus, we explored whether AMBRA1 promotes NRF2 degradation by modulating the KEAP1‐dependent ubiquitination of NRF2. AMBRA1 overexpression did not affect KEAP1 expression in HT29 or HIEC‐6 cells or the interaction between KEAP1 and NRF2 (Figure S2B,C, Supporting Information). In addition, KEAP1 knockdown did not abolish the decreased protein level or increased ubiquitination level of NRF2 induced by AMBRA1 overexpression, suggesting that AMBRA1 may facilitate NRF2 degradation in a KEAP1‐independent manner (Figure 2F,G). Cys273 and Cys288 are crucial for the KAEP1‐mediated ubiquitination and degradation of NRF2, and the C273/288A KEAP1 mutant acts as a dominant‐negative mutant that abrogates KEAP1‐mediated degradation of NRF2.^[^ ^27^ ^]^ Consistent with the results of KEAP1 knockdown, the KEAP1‐C273/288A mutation had no effect on the downregulated protein level or upregulated ubiquitination level of NRF2 induced by AMBRA1 overexpression (Figure 2H,I). Notably, we observed that AMBRA1 overexpression further enhanced the KEAP1‐mediated degradation and ubiquitination of NRF2, indicating an additive effect of AMBRA1 on NRF2 degradation (Figure 2J,K). Collectively, these data demonstrated that AMBRA1 promotes K48‐linked polyubiquitination mediated‐NRF2 degradation through a pathway that is likely parallel to the KEAP1‐NRF2 pathway.

### 2.3 AMBRA1 Inhibits DUB3‐Mediated NRF2 Deubiquitination and Stabilization

**Figure 3:**
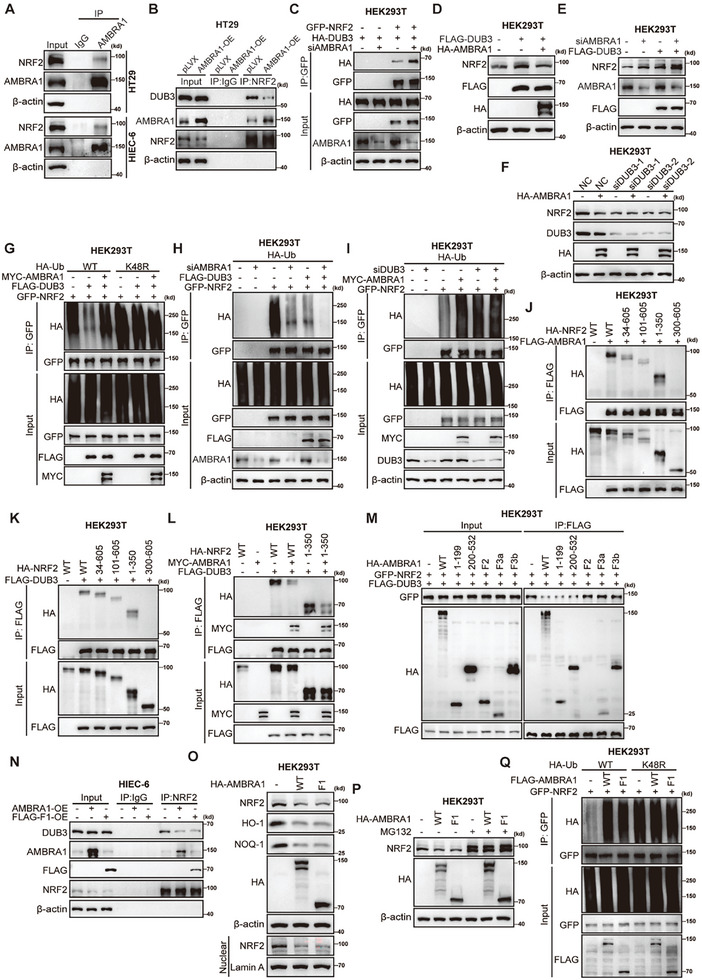
The N‐term F1 domain is required for AMBRA1 to inhibit the DUB3‐mediated NRF2 deubiquitination. A) Endogenous co‐IP of AMBRA1 and NRF2 in HT29 and HIEC‐6 cells. B) Endogenous co‐IP of NRF2 and DUB3 in control and AMBRA1‐overexpressing HT29 cells. C) Exogenous co‐IP of GFP‐NRF2 and HA‐DUB3 in HEK293T cells transfected with control or siRNA targeting AMBRA1. D) HEK293T cells were transfected with the indicated control vector or the indicated FLAG‐DUB3 or HA‐AMBRA1 plasmids. NRF2 expression was measured via western blotting. E) HEK293T cells were transfected with control and siRNA targeting AMBRA1 and indicated control and FLAG‐DUB3 plasmids. NRF2 expression was examined via western blotting. F) HEK293T cells were transfected with control or siRNA targeting DUB3 and the indicated control or HA‐AMBRA1 plasmids. NRF2 expression was measured via western blotting G) HEK293T cells were transfected with the indicated HA‐Ub, HA‐Ub‐K48R, GFP‐NRF2, MYC‐AMBRA1 and FLAG‐DUB3 plasmids. Lysates of the cells were used for immunoprecipitation with an anti‐GFP antibody for subsequent quantification of NRF2 ubiquitination via western blotting. H) The control and AMBRA1‐knockdown HEK293T cells were transfected with HA‐Ub, FLAG‐DUB3 and GFP‐NRF2 plasmids. The cell lysates were used for immunoprecipitation with an anti‐GFP antibody for subsequent quantification of NRF2 ubiquitination via western blotting. I) The control and DUB3‐knockdown HEK293T cells were transfected with HA‐Ub, GFP‐NRF2 and MYC‐AMBRA1 plasmids. The cell lysates were used for immunoprecipitation with an anti‐GFP antibody for subsequent quantification of NRF2 ubiquitination via western blotting. J,K) Exogenous co‐IP of FLAG‐AMBRA1, FLAG‐DUB3 and full‐length/truncated HA‐NRF2 in HEK293T cells. L) Exogenous co‐IP of FLAG‐DUB3 and full‐length HA‐NRF2 and 1–350 truncation of HA‐NRF2 with or without coexpression of MYC‐AMBRA1. M) Exogenous co‐IP of FLAG‐DUB3 and GFP‐NRF2 in HEK293T cells with the co‐transfection of full‐length/truncated HA‐AMBRA1. N) Endogenous co‐IP of NRF2 and DUB3 in pLVX‐vector control, AMBRA1‐overexpressing and F1 domain‐overexpressing HIEC‐6 cells. O) HEK293T cells were transfected with the indicated control, full‐length and the F1 fragment of HA‐AMBRA1 plasmids. The total protein levels of NRF2, HO‐1 and NQO‐1 and the nuclear expression level of NRF2 were determined via western blotting. P) The protein levels of NRF2 were measured in HEK293T cells transfected with control, full‐length/F1 fragment HA‐AMBRA1 and treated with or without MG132 (10 µM) via western blotting. Q) HEK293T cells were transfected with the indicated HA‐Ub, GFP‐NRF2, FLAG‐AMBRA1 and FLAG‐AMBRA1‐F1 fragment plasmids. Lysates of the cells were used for immunoprecipitation with an anti‐GFP antibody for subsequent quantification of NRF2 ubiquitination via western blotting.

To explore whether AMBRA1 directly modulates NRF2 expression, we performed reciprocal exogenous and semi‐endogenous co‐immunoprecipitation (co‐IP) and observed that AMBRA1 interacted with NRF2 in HEK293T cells (Figure S3A,B, Supporting Information). Endogenous co‐IP confirmed the interaction between AMBRA1 and NRF2 in HT29 and HIEC‐6 cells (**Figure** **3**A).

To explore the nature of the AMBRA1/NRF2 interaction, we used various truncated AMBRA1 proteins produced in our recent study.^[^ ^23^ ^]^ We found that only the AMBRA1 protein with the C‐terminal truncation failed to interact with NRF2, suggesting that AMBRA1 interacted with NRF2 through multiple interfaces (Figure S3C, Supporting Information). A recent study reported that DUB3 stabilizes NRF2 via deubiquitination of NRF2.^[^ ^17^ ^]^ Notably, the interaction between DUB3 and NRF2 was dramatically diminished by AMBRA1 overexpression in HEK293T cells (Figure S3D,E, Supporting Information). A similar result was observed in HT29 cells via endogenous co‐IP analysis of the DUB3/NRF2 interaction (Figure 3B). We also found that knockdown of AMBRA1 markedly enhanced the interaction between NRF2 and DUB3 (Figure 3C). Consistent with these observations, AMBRA1 overexpression attenuated NRF2 deubiquitination and stabilization by DUB3, whereas AMBRA1 knockdown promoted the DUB3‐mediated deubiquitination and stabilization of NRF2 (Figure 3D,E,G,H). Importantly, the elevated polyubiquitination and downregulated protein levels of NRF2 caused by AMBRA1 overexpression were abrogated by DUB3 knockdown, suggesting that AMBRA1 promotes NRF2 degradation by modulating DUB3 (Figure 3F,I).

Next, we explored the nature of interactions between NRF2 and AMBRA1/DUB3. By generating and using a series of truncated forms of NRF2, we found that both AMBRA1 and DUB3 interacted with the N‐terminal fragment of NRF2 (amino acid residues 1–350) (Figure 3J,K). Notably, AMBRA1 decreased the interaction between DUB3 and both full‐length NRF2 and the N‐terminal fragment of NRF2, indicating that it competed with DUB3 for interaction with the N‐terminal region of NRF2 (Figure 3L). Although multiple domains of AMBRA1 mediate the AMBRA1/NRF2 interaction, we surprisingly observed that only the N‐terminal truncated forms of AMBRA1 (amino acid residues 1–199 and 200–532, which together comprise the F1 domain) diminished the interaction between DUB3 and NRF2, and that their effect was comparable to that of full‐length AMBRA1 (Figure 3M). Moreover, both exogenous and semi‐endogenous co‐IP revealed that the inhibitory effect of the AMBRA1 F1 domain on the DUB3/NRF2 interaction was comparable to that of full‐length WT AMBRA1 (Figure S3F,G, Supporting Information). Endogenous co‐IP experiments with HIEC‐6 cells also showed that the F1 domain was sufficient to disassociate the DUB3/NRF2 complex (Figure 3N; Figure S3H, Supporting Information). Consistent with the effect of the AMBRA1 F1 domain on the DUB3/NRF2 interaction, transient transfection of HEK293T cells with a vector expressing the F1 domain decreased the total and nuclear protein levels of NRF2, which was abolished by MG132 treatment (Figure 3O,P). The overexpression of the AMBRA1 F1 domain drastically promoted NRF2 ubiquitination (Figure 3Q). Overall, these results indicate that the N‐terminal F1 domain of AMBRA1 is required to antagonize the interaction between DUB3 and NRF2, thereby suppressing DUB3‐dependent NRF2 deubiquitination and promoting NRF2 protein degradation.

### 2.4 USP7 Stabilizes the AMBRA1 Protein

**Figure 4:**
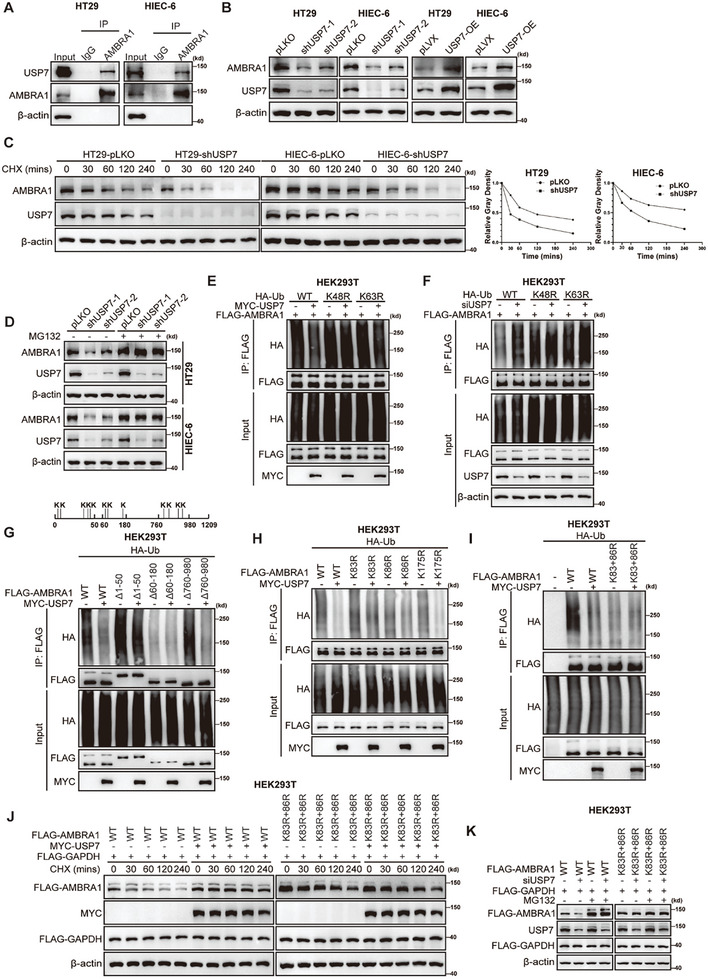
USP7 stabilizes the AMBRA1 protein. A) Endogenous co‐IP of AMBRA1 and USP7 in HT29 and HIEC‐6 cells. B) Western blot analysis of AMBRA1 expression in USP7‐knockdown and USP7‐overexpressing HT29 and HIEC‐6 cells. C) Analysis of the half‐life of the AMBRA1 protein in control and USP7‐knockdown HT29 and HIEC‐6 cells. The cells were treated with cycloheximide (CHX) (75 µg ml^−1^) for the indicated times prior to western blot analysis of AMBRA1 expression. D) The protein levels of AMBRA1 were measured in control and USP7‐knockdown HT29 and HIEC‐6 cells with or without MG132 (10 µM) treatment via western blotting. E) HEK293T cells were transfected with HA‐Ub, HA‐Ub‐K48R, HA‐Ub‐K63R, FLAG‐AMBRA1 and MYC‐USP7. Cell lysates were used for immunoprecipitation of FLAG‐AMBRA1 and subsequent quantification of the ubiquitination level via western blotting. F) The control and USP7‐knockdown HEK293T cells were transfected with HA‐Ub, HA‐Ub‐K48R, HA‐Ub‐K63R and FLAG‐AMBRA1. FLAG‐AMBRA1 was immunoprecipitated for subsequent quantification of the ubiquitination level of AMBRA1 via western blotting. G) HEK293T cells were transfected with HA‐Ub, FLAG‐AMBRA1 WT, Δ1‐50, Δ60‐180, Δ760‐980 and MYC‐USP7. Cell lysates were used for immunoprecipitation of FLAG‐AMBRA1 and subsequent quantification of the ubiquitination level via western blotting. H) HEK293T cells were transfected with HA‐Ub, FLAG‐AMBRA1^WT^, FLAG‐AMBRA1^K83R^, FLAG‐AMBRA1^K86R^, FLAG‐AMBRA1^K175R^ and MYC‐USP7. Cell lysates were used for immunoprecipitation of FLAG‐AMBRA1 and subsequent quantification of the ubiquitination level via western blotting. I) HEK293T cells were transfected with HA‐Ub, FLAG‐AMBRA1^WT^, FLAG‐AMBRA1^K83R+86R^ and MYC‐USP7. The ubiquitination level was analyzed as described above. J) Analysis of the half‐life of the WT and K83R+86R mutant FLAG‐AMBRA1 in HEK293T cells transfected with control and MYC‐USP7 plasmids. Cells were treated with CHX (75 µg ml^−1^) for the indicated times before western blot analysis. K) The protein levels of the WT and K83R+86R mutant FLAG‐AMBRA1 were measured via western blotting in HEK293T cells transfected with control and MYC‐USP7 plasmids with or without MG132 (10 µM) treatment.

Identification of DUB3 as an important deubiquitinase that acts on NRF2 prompted us to explore the mechanisms underlying AMBRA1 deubiquitination and stabilization. We performed immunoprecipitation‒mass spectrometry (IP‐MS) analysis of HT29 cells stably expressing FLAG‐AMBRA1 and found that the deubiquitinating enzyme, USP7, could interact with AMBRA1 (Table S1, Supporting Information). Whereas IP‐MS analysis did not reveal that USP15 could be a potential candidate for interacting with AMBRA1. Thus, USP7 was selected in this study to explore whether USP7 could regulate AMBRA1 protein expression. We confirmed the interaction between AMBRA1 and USP7 in HEK293T cells via reciprocal exogenous and semi‐endogenous co‐IP (Figure S4A,B, Supporting Information). Endogenous co‐IP confirmed the interaction between AMBRA1 and USP7 in HT29 and HIEC‐6 cells (**Figure** **4**A). A domain mapping assay revealed that the F1, F2, and F3b domains of AMBRA1, but not the F3a domain, interacted with USP7; however, only the N‐terminal 1–206 truncation of USP7 mediated this interaction with AMBRA1 (Figure S4C,D, Supporting Information).

Next, we explored whether USP7 regulates the stability of AMBRA1. As expected, USP7 knockdown significantly decreased AMBRA1 protein levels, whereas USP7 overexpression markedly upregulated AMBRA1 expression in HT29 and HIEC‐6 cells (Figure 4B). CHX assays revealed that the half‐life of endogenous AMBRA1 was shorter in HT29 and HIEC‐6 cells with USP7 knockdown than in control cells (Figure 4C). Furthermore, the effect of USP7 knockdown on AMBRA1 protein levels was abolished by MG132 treatment (Figure 4D). The results of the ubiquitin assay indicated that USP7 markedly decreased the K48‐linked polyubiquitination of AMBRA1, and that USP7 knockdown increased it (Figure 4E,F). Similar results were observed when the USP7 inhibitor, P5091, was used (Figure S5A–C, Supporting Information). To strengthen these data, we constructed expression plasmids encoding catalytically inactive mutants of USP7; these were USP7 (C223S) and the Δ206‐560 domain (the hand‐like catalytic domain). In HEK293T cells, transient knockdown of USP7 also markedly decreased AMBRA1 expression, whereas overexpression of USP7 increased it; however, these changes did not occur when the catalytically inactive mutation (C223S) or the mutant lacking the catalytic domain (Δ206‐560) was expressed (Figure S4E–G, Supporting Information). Consistent with the above observations, the C223S and Δ206‐560 mutants of USP7 failed to downregulate the K48‐ubiquitination level of AMBRA1 (Figure S4H,I, Supporting Information).

Next, we determined the specific sites at which AMBRA1 is deubiquitinated by USP7. To this end, we generated a series of truncated forms of AMBRA1 containing subsets of potential ubiquitination sites^[^ ^28^ ^]^ and measured the ubiquitination levels of these truncations after co‐transfection with USP7 in HEK293T cells. Interestingly, the Δ60‐180 truncation showed minimal ubiquitination and was not deubiquitinated after cotransfection with USP7, whereas the other two truncations (Δ1‐50 and Δ760‐980) were deubiquitinated when the cells were cotransfected with MYC‐USP7 (Figure 4G). These results indicate that the three lysine residues (K83, K86, and K175) in the 60–180 region of AMBRA1 are the main ubiquitination sites in AMBRA1. To further determine the sites at which AMBRA1 is deubiquitinated by USP7, we constructed several AMBRA1 mutants: K83R, K86R, and K175R. We found that USP7 induced partial deubiquitination of the AMBRA‐K83R and AMBRA‐K86R mutants, but failed to affect AMBRA1 ubiquitination in the K83R/K86R double mutant (Figure 4H,I). In addition, USP7 overexpression stabilized WT AMBRA1 but not K83R/K86R mutant AMBRA1 in HEK293T cells (Figure 4J). Notably, the K83R/K86R double mutation only partially attenuated the degradation of the AMBRA1 protein, and neither USP7 knockdown nor MG132 treatment affected the expression of the mutant protein, suggesting that the K83R/K86R double mutation affected only the proteasomal degradation of AMBRA1 (Figure 4J,K). Taken together, these data indicate that USP7 stabilizes the AMBRA1 protein by deubiquitinating it at lysine residues 83 and 86.

### 2.5 AMBRA1 Protein Levels are Increased upon H_2_O_2_ Stimulation in IECs

**Figure 5:**
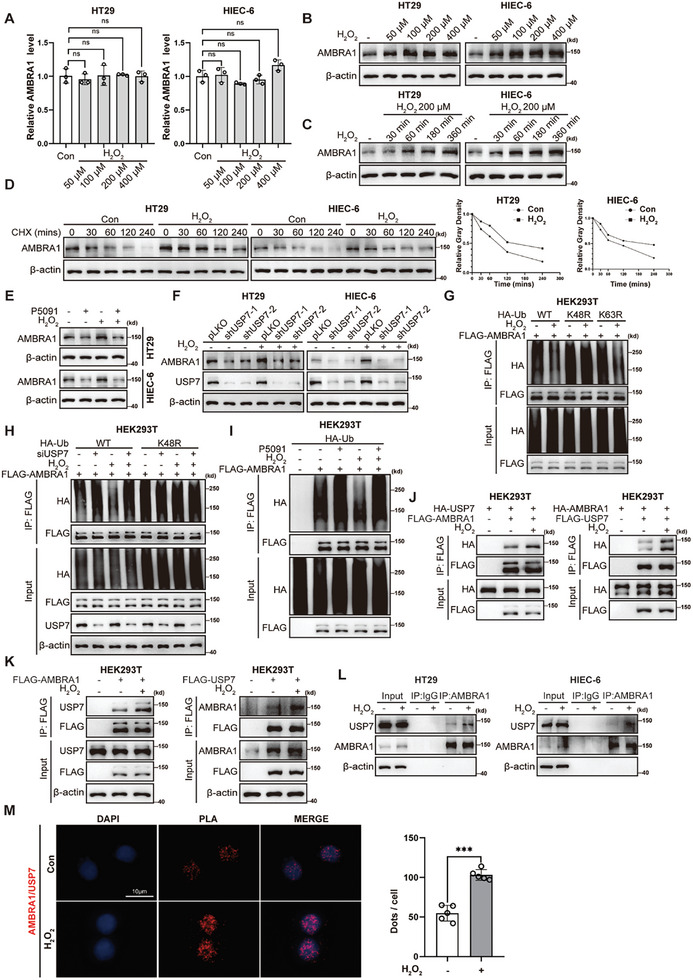
H_2_O_2_ stimulation increases AMBRA1 protein levels in IECs. A) qRT‒PCR analysis of AMBRA1 mRNA levels in HT29 and HIEC‐6 cells treated with H_2_O_2_ (50, 100, 200 or 400 µM) for 6 h. B) Western blot analysis of AMBRA1 expression in HT29 and HIEC‐6 cells treated with H_2_O_2_ (50, 100, 200 or 400 µM) for 6 h. C) Western blot analysis of AMBRA1 expression in HT29 and HIEC‐6 cells treated with H_2_O_2_ (200 µM) for the indicated times. D) Analysis of the half‐life of AMBRA1 protein in HT29 cells with or without H_2_O_2_ (200 µM) treatment. The cells were treated with CHX (75 µg ml^−1^) for the indicated times before western blot analysis of AMBRA1 expression. E) Western blot analysis of AMBRA1 expression in HT29 and HIEC‐6 cells treated with H_2_O_2_ (200 µM) alone or in combination with P5091 (10 µM) for the indicated times. F) Western blot analysis of AMBRA1 expression in USP7‐knockdown and control HT29 and HIEC‐6 cells treated with H_2_O_2_ (200 µM). G) HEK293T cells were transfected with the HA‐Ub, HA‐Ub‐K48R, HA‐Ub‐K63R, FLAG‐AMBRA1 plasmids and treated with or without H_2_O_2_ (200 µM). The cell lysates were used for immunoprecipitation with an anti‐FLAG antibody for subsequent quantification of AMBRA1 ubiquitination via western blotting. H) The control and USP7‐knockdown HEK293T cells were transfected with HA‐Ub, FLAG‐AMBRA1 plasmids and treated with or without H_2_O_2_ (200 µM). The cell lysates were used for immunoprecipitation with an anti‐ FLAG antibody for subsequent quantification of AMBRA1 ubiquitination via western blotting. I) HEK293T cells were transfected with the HA‐Ub, FLAG‐AMBRA1 plasmids and treated with H_2_O_2_ (200 µM) alone or in combination with P5091 (10 µM). The ubiquitination level of AMBRA1 was analyzed as described above. J) Exogenous co‐IP of FLAG‐AMBRA1 and HA‐USP7, FLAG‐USP7 and HA‐AMBRA1 in HEK293T cells treated with or without H_2_O_2_ stimulation (200 µM). K) Semi‐endogenous co‐IP of exogenous FLAG‐AMBRA1 and FLAG‐USP7 and endogenous USP7 and AMBRA1 in HEK293T cells treated with or without H_2_O_2_ stimulation (200 µM). L) Endogenous co‐IP of AMBRA1 and USP7 in HT29 and HIEC‐6 cells with or without H_2_O_2_ stimulation (200 µM). M) In situ PLA (red foci) analysis of the interaction between AMBRA1 and USP7 in H_2_O_2_‐treated (200 µM) and control HT29 cells. Scale bars, 10 µm. The data are presented as the means ± SDs. n = 3 (A) biologically independent samples per group. These data (A, M) are representative of 2 independent experiments. n = 5 (M) technical replicates per group. One‐way ANOVA (A) and students’ t test (M) were performed to assess statistical significance. *** *P* < 0.001.

Previously, we showed that AMBRA1 is stabilized when IECs are stimulated by TNF‐α.^[^ ^23^ ^]^ Therefore, we investigated whether AMBRA1 was modulated by intestinal oxidative stress. Interestingly, we found that the mRNA level of AMBRA1 was not significantly altered by H_2_O_2_ stimulation, whereas AMBRA1 protein levels in HT29 and HIEC‐6 cells were upregulated by H_2_O_2_ treatment in a dose‐ and time‐dependent manner (**Figure** **5**A–C). Furthermore, endogenous AMBRA1 in H_2_O_2_‐treated HT29 and HIEC‐6 cells had an increased half‐life, according to the CHX assay (Figure 5D). The H_2_O_2_‐induced upregulation of AMBRA1 protein levels in HT29 and HIEC‐6 cells was largely abrogated by treatment with the USP7 inhibitor P5091 and USP7 knockdown (Figure 5E,F). H_2_O_2_ stimulation consistently inhibited K48‐linked polyubiquitination of AMBRA1, and this effect was abrogated in USP7 knockdown cells and in cells treated with P5091 (Figure 5G,I). These results suggest that the H_2_O_2_‐induced upregulation of AMBRA1 protein levels may be dependent on USP7.

Intriguingly, reciprocal exogenous and semiendogenous co‐IP revealed that the interaction between AMBRA1 and USP7 was enhanced after H_2_O_2_ stimulation of HEK293T cells (Figure 5J,K). Endogenous co‐IP further demonstrated that H_2_O_2_ treatment facilitated the formation of the AMBRA1/USP7 complex in HT29 and HIEC‐6 cells (Figure 5L). The proximity ligation assay (PLA) results further confirmed that the interaction between endogenous AMBRA1 and USP7 was enhanced upon H_2_O_2_ stimulation in HT29 cells (Figure 5M). Notably, the PLA signal produced by the AMBRA1/USP7 interaction was observed mainly in the nucleus, suggesting that the interaction between the two proteins occurred in the nucleus (Figure 5M). Collectively, these results demonstrate that oxidative stress stabilizes AMBRA1 by enhancing its binding to USP7 and the subsequent USP7‐mediated AMBRA1 deubiquitination.

### 2.6 Specific USP7 Inhibitor P5091 Inhibits Oxidative Stress and Attenuates DSS‐Induced Colitis in Vivo

**Figure 6:**
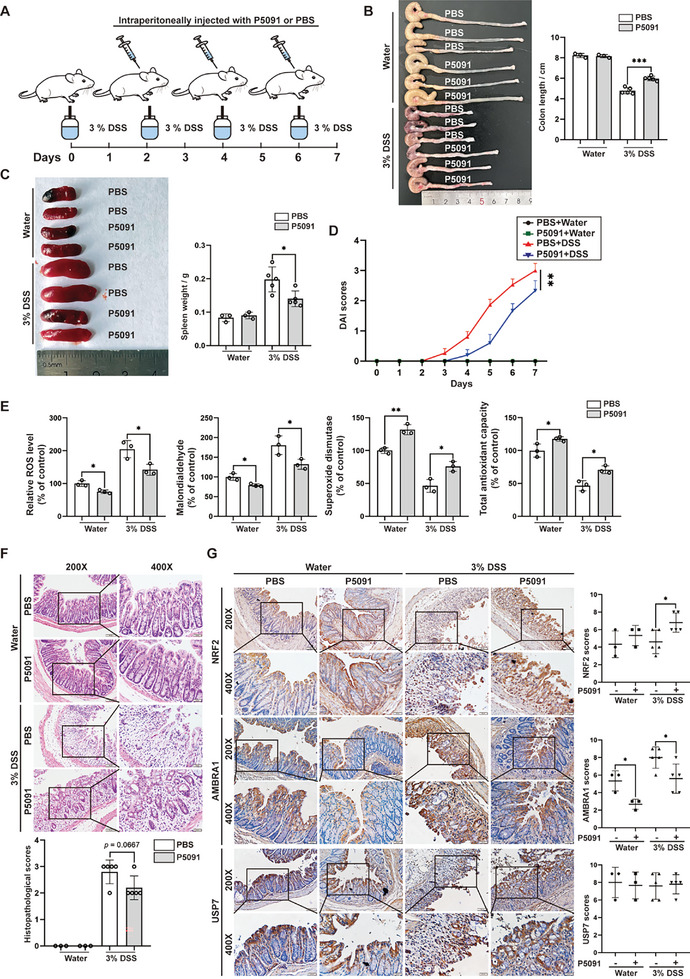
USP7 inhibitor P5091 diminishes oxidative stress and attenuates DSS‐induced colitis in vivo. A) Schematic diagram of the P5091 treatment in the DSS‐induced acute colitis model. C57BL/6 mice were administered 3% DSS and treated with or without P5091 (20 mg k^−1^g) on Day 2, 4, and 6 during colitis induction (n = 3 for control group, n = 5 for DSS‐treated group). B,C) Representative images and statistical analysis of the colorectum lengths and spleen weights of control and P5091‐treated mice. D) The loss of body weight and the severity of diarrhoea and bleeding, as evaluated by the DAI score, were determined in control and DSS‐induced mice with or without P5091 treatment. E) The levels of oxidative stress markers (ROS, T‐AOC, SOD and MDA) were examined in colon tissues from control and DSS‐induced mice with or without P5091 treatment. F) The pathological scores of colons from control and DSS‐induced mice with or without P5091 treatment were evaluated via hematoxylin–eosin staining (magnification: upper panels, 200×; lower panels, 400×). G) The expression of NRF2, AMBRA1 and USP7 were examined in control and P5091‐treated mice via immunohistochemistry. The data are presented as the means ± SDs. n = 3 biologically independent samples for control group, n = 5 biologically independent samples for DSS group (B–D, F, G). n = 3 (E) biologically independent samples per group. One‐way ANOVA (B, C, E), two‐way ANOVA (D) and the Mann‒Whitney's U test (F, G) were performed to assess statistical significance. * *P* < 0.05, ** *P* < 0.01, *** *P* < 0.001.

Our in vitro assays revealed that P5091 significantly enhanced the interaction between DUB3 and NRF2 and inhibited K48‐linked NRF2 ubiquitination, resulting in increased expression of NRF2, HO‐1, and NQO1 in P5091‐treated HT29 cells (Figure S5D–F, Supporting Information). Thus, we speculated that the inhibition of USP7 could lead to the downregulation of AMBRA1 expression, reducing the competitive binding of AMBRA1 to NRF2 and enhancing DUB3‐mediated NRF2 deubiquitination, thereby alleviating intestinal inflammation. To test this hypothesis, we examined the antioxidative effects of P5091 on mice with DSS‐induced acute colitis (**Figure** **6**A). As shown in Figure 6B–D, P5091‐treated mice with DSS‐induced colitis presented with alleviated colitis, which manifested as longer colon length, lower spleen weight, and lower disease activity index (DAI) scores than those treated with PBS. Analysis of oxidative stress markers showed that mice treated with P5091 presented lower levels of ROS and MDA but higher levels of SOD and T‐AOC than untreated mice (Figure 6E). Furthermore, DSS‐induced mice treated with P5091 showed less histopathological destruction, lower levels of AMBRA1 expression, and higher levels of NRF2, HO‐1, and NQO‐1 proteins than mice that did not receive P5091 (Figure 6F,G, Figure S6A,B, Supporting Information). Collectively, these results demonstrate that the USP7 inhibitor P5091 inhibits oxidative stress and attenuates DSS‐induced colitis in vivo.

We further explored the protective effects of P5091 on intestinal barrier integrity during intestinal inflammation. Alcian blue/periodic acid‐Schiff staining revealed that the administration of P5091 significantly attenuated the decrease in the number of goblet cells in mice with DSS‐induced acute colitis (Figure S7A, Supporting Information). Consistently, MUC2 expression in DSS‐treated mice was moderately upregulated by P5091 (Figure S7B, Supporting Information). Immunofluorescence staining of epithelial cell markers such as ZO‐1, CK20, and E‐cadherin revealed less damage to the intestinal barrier after inflammatory injury in mice treated with P5091 (Figure S7C–E, Supporting Information). Collectively, these results suggest that USP7 inhibition is a potential therapeutic strategy for attenuating intestinal oxidative stress and ameliorating colitis.

### 2.7 AMBRA1 is Highly Expressed in Inflamed Colons and is Negatively Correlated with NRF2 in the Clinic

**Figure 7:**
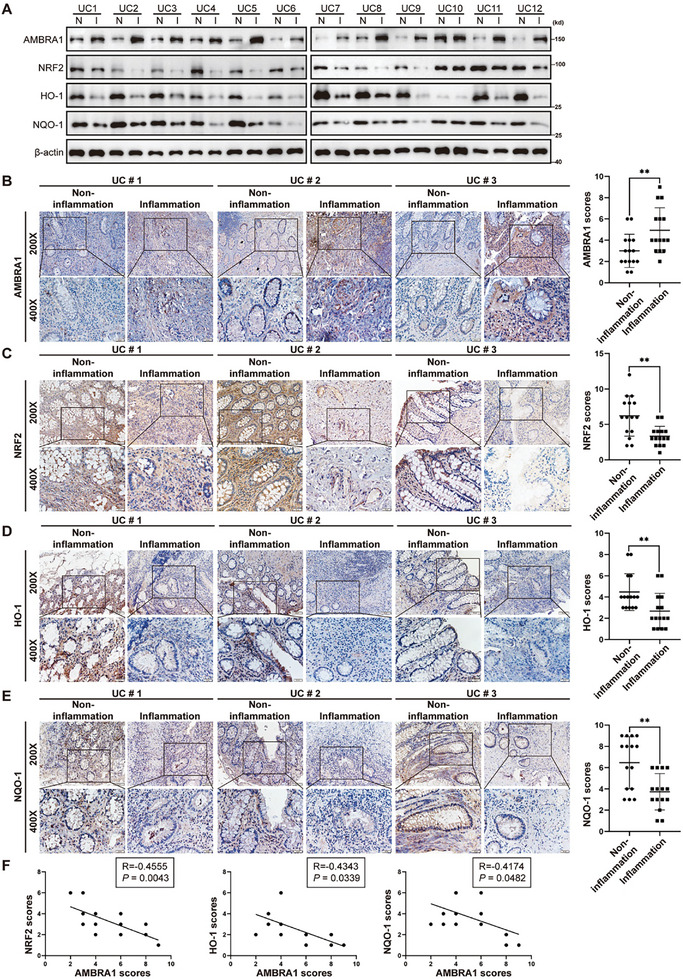
AMBRA1 is highly expressed in inflamed colon, and its expression is negatively correlated with that of NRF2. A) AMBRA1, NRF2, HO‐1 and NQO‐1 expression levels were examined in inflamed and paired adjacent non‐inflamed colon tissues from UC patients via western blotting (n = 12). B–E) AMBRA1 (B), NRF2 (C), HO‐1 (D) and NQO‐1 (E) protein levels were examined in inflamed and paired adjacent non‐inflamed colon tissues from UC patients via immunohistochemistry (n = 15). F) Spearman's correlation analysis was used to evaluate the correlations between the protein level of AMBRA1 and the levels of NRF2, HO‐1 and NQO‐1 in inflamed colon tissues from patients with UC. The data are presented as the means ± SDs. n = 12 (A), n = 15 (B–F) biologically independent samples per group. The Wilcoxon signed‐rank test (B‐E) and Spearman's correlation analysis (F) were performed to assess statistical significance. **P < 0.01.

To determine whether AMBRA1 expression correlated with the expression of NRF2, HO‐1 and NQO‐1, we first examined the expression of these proteins in inflamed and noninflamed intestinal tissues from patients with UC. We recently reported that the expression of AMBRA1 is moderately to dramatically increased in inflamed colon tissue.^[^ ^23^ ^]^ Conversely, the expression levels of NRF2, HO‐1 and NQO‐1 were moderately decreased in inflamed colon tissues (**Figure** **7**A–E). Furthermore, Spearman's correlation analysis revealed that immunohistochemical scores for AMBRA1 were negatively correlated with immunohistochemical scores for NRF2 (R = – 0.4555, p = 0.0043), HO‐1 (R = ‐0.4343, p = 0.0339) and NQO‐1 (R = ‐0.4174, p = 0.0482) (Figure 7F). Taken together, these results indicate that high AMBRA1 expression in inflamed colons is negatively correlated with the expression of the antioxidative proteins NRF2, HO‐1, and NQO‐1, and could play a role in inducing oxidative stress and the development of IBD.

## 3 Discussion

AMBRA1, a multifunctional scaffold protein that is a well‐known pro‐autophagic protein, plays a crucial role in various biological processes, including apoptosis, cell proliferation and cell invasion.^[^ ^20^ ^]^ We recently reported that AMBRA1 promotes intestinal epithelial inflammation by antagonizing PP4R1/PP4c‐mediated IKK dephosphorylation in an autophagy‐independent manner and demonstrated the proinflammatory function and autophagy‐independent mechanism of action of AMBRA1 during the development and progression of IBD.^[^ ^23^ ^]^ In this study, we discovered that AMBRA1 acts as a competitive binding protein and an antagonist of the transcription factor NRF2, thus functioning as a pro‐oxidative factor during intestinal inflammation, in addition to its role in autophagy.

IBD is associated with oxidative stress and the adaptive NRF2‐driven response of colonic epithelial cells to OS during chronic intestinal inflammation.^[^ ^8^, ^9^, ^11^ ^]^ Both increased and decreased expression of NRF2 in intestinal tissues has been reported in patients with IBD, suggesting that a disruption of physiological redox signaling is also associated with the onset and progression of IBD.^[^ ^29^, ^30^, ^31^ ^]^ Nevertheless, higher levels of NRF2 are positively correlated with antioxidative enzymes and negatively correlated with the expression of the proinflammatory cytokine IL‐17A in IBD.^[^ ^31^ ^]^ Similar to clinical reports, we did not observe significant upregulation of NRF2 protein level in our DSS‐induced colitis mouse model. The expression of NRF2 was also slightly increased in LPS‐induced mouse model of chronic renal inflammation and showed no significant change in cavernous nerve injury‐induced oxidative stress rat model.^[^ ^32^, ^33^ ^]^ NRF2 is an unstable protein that can be stabilized by redox stimulation in a short time, especially in cell line‐based experiments in vitro. The different degrees of change in NRF2 protein levels in vitro and in vivo could be due to the dynamic regulation of NRF2 during oxidative stress, probably involving both positive and negative feedback loops for modulating NRF2 in response to redox stimulation.^[^ ^34^, ^35^, ^36^ ^]^ Thus, NRF2 levels were not consistently or significantly upregulated, especially after exposure to long‐term inflammatory and oxidative stress in *vivo*. Nevertheless, we found that the loss of AMBRA1 by gene knockout in IECs or protein destabilization using a USP7 inhibitor led to significant upregulation of NRF2, which further supports that AMBRA1 functions as a pro‐oxidative factor by promoting NRF2 degradation. Our finding that high AMBRA1 expression is negatively correlated with levels of NRF2 and its related antioxidative enzymes in IBD clinical samples demonstrates that upregulation of AMBRA1 promotes intestinal inflammation not only through IKK dephosphorylation and subsequent activation of NF‐κB activity but also through perturbation of physiological NRF2‐redox signaling and increased oxidative stress. Notably, NRF2 can impede NF‐ĸB activity through multiple mechanisms;^[^ ^37^ ^]^ thus, AMBRA1 could also activate NF‐ĸB by indirectly suppressing NRF2 expression.

In addition to the canonical KEAP1‐dependent protein degradation, NRF2 protein stability can also be modulated by other E3 ligases and deubiquitinase in different contexts or disease conditions. GSK‐3β mediated phosphorylation of NRF2 also promotes NRF2 degradation via the adaptor protein β‐TrCP‐dependent E3 ligase complex, independent on KEAP1.^[^ ^13^ ^]^ The GSK‐3β/NRF2 regulatory axis plays a vital role in mediating electroacupuncture antioxidative effects, modulating ferroptosis, hindering acute kidney injury to chronic kidney disease transition and protecting pancreatic beta cells from oxidative stress.^[^ ^38^, ^39^, ^40^, ^41^ ^]^ Recently, a new β‐TrCP protein isoform, encoded by a circular RNA circ‐β‐TrCP, was revealed to competitively bind NRF2 and block SCF^β‐TrCP^‐mediated NRF2 proteasomal degradation in trastuzumab resistant breast cancer, which further implicates the important physiologic and pathological role of NRF2 modulation independent on KEAP1.^[^ ^42^ ^]^ In this study, we discovered a novel mechanism of NRF2 degradation in IBD and demonstrated that AMBRA1, an adaptor protein of the CUL4‐RING E3 ubiquitin ligase, promoted NRF2 degradation in a KEAP1‐independent manner. Notably, AMBRA1 enhanced NRF2 degradation by antagonizing the interaction between NRF2 and its newly identified deubiquitinase, DUB3, but did not function as an E3 ligase of NRF2. It is worth noting that the N‐terminal F1 domain of AMBRA1 was sufficient to competitively interact with NRF2 and disrupt the NRF2‐DUB3 complex. AMBRA1 functions as an adaptor protein of the DDB1‐Cullin4 E3 ligase via the DDB1‐binding site, which is also located at the N‐term.^[^ ^20^ ^]^ Thus, we speculated that the interaction between AMBRA1 and NRF2 blocked the binding site for DDB1 and the recruitment of the DDB1‐Cullin4 E3 ligase. Recently, a second deubiquitinase of NRF2 was identified that OTUD1 stabilizes NRF2 to ameliorate hepatic ischemia/reperfusion injury by both inhibiting NRF2‐KEAP1 binding and directly deubiquitinating NRF2.^[^ ^43^ ^]^ Similar to the dual pro‐inflammatory and pro‐oxidative roles of AMBRA1, OTUD1 possesses dual anti‐inflammatory and anti‐oxidative roles by hydrolyzing the K63‐linked ubiquitin chains from NF‐κB signaling factors and NRF2.^[^ ^44^, ^45^ ^]^ These findings suggest that both AMBRA1 and OTUD1 are potential targets for the treatment of IBD because of their dual regulatory functions in inflammation and the oxidative stress response.

As a key autophagic‐associated protein, AMBRA1 is dynamically regulated during the initiation of autophagy and upon inflammatory stimulation.^[^ ^20^, ^21^ ^]^ In particular, autophagic stimuli and TNF‐α trigger AMBRA1 phosphorylation, leading to dissociation of AMBRA1 from CUL4‐DDB1 E3 ligase and increased protein stability.^[^ ^46^ ^]^ Additionally, previous studies reported that RNF2 ubiquitylated AMBRA1 at Lys45 with Lys48‐linked ubiquitin chains to suppression of autophagy^[^ ^28^ ^]^ and IKKα kinase phosphorylated AMBRA1 on its serine 1043 to induce its structural changes and mitophagic activity.^[^ ^47^ ^]^ Similarly, our recent research reported that S1043 phosphorylation of AMBRA1 by IKKα led to a dissociation of AMBRA1‐CUL4A interaction and consequent increased AMBRA1 protein levels in IECs under inflammation.^[^ ^23^ ^]^ As mentioned above, the post‐translational modifications, mainly including the phosphorylation and ubiquitination modification of AMBRA1, could be crucial for its stability. However, the regulatory mechanism of AMBRA1 stability in intestinal oxidative stress remains unclear. Interestingly, we found that the mRNA level of AMBRA1 was not significantly altered, whereas the AMBRA1 protein levels in IECs were upregulated by H_2_O_2_ treatment, which indicated that AMBRA1 protein stabilization is induced by oxidative stress independent on AMBRA1 gene transcription. Oxidative stress can promote the interaction between AMBRA1 and its deubiquitinase USP7, thereby reducing the polyubiquitination level of AMBRA1 K83/K86 and enhancing AMBRA1 protein stability. It has been reported that phosphorylation of AMBRA1 could induce structural changes in the AMBRA1 protein that promote its interaction with LC3 upon mitophagy stimulation.^[^ ^47^ ^]^ Moreover, a recent study identified a covalent allosteric site, Cys576, as a novel site for USP7 inhibition.^[^ ^15^ ^]^ Since H_2_O_2_ induces oxidative modifications of cysteine and histidine residues in proteins,^[^ ^48^ ^]^ we speculated that AMBRA1 or USP7 might undergo oxidative modification and that such modifications may lead to structural changes in proteins and complex formation. This should be explored in future studies on the biochemical and structural nature of the AMBRA1‐USP7 complex. Notably, the AMBRA1‐USP7 complex exists mainly in the nucleus, indicating that USP7 deubiquitinates and stabilizes nuclear AMBRA1. Indeed, USP7 is primarily nuclear and is known as a nuclear deubiquitinase that modulates various transcription factors, such as p53.^[^ ^49^ ^]^ Recent studies have demonstrated the nuclear localization of AMBRA1 and its potential role in gene transcription.^[^ ^50^, ^51^ ^]^ Given that NRF2 is a transcription factor that functions in the nucleus, we believe that the nuclear localization of AMBRA1 could be vital for its competitive binding to NRF2. In addition, the E3 ligase RNF2 catalyzes the K48‐linked polyubiquitination of AMBRA1 at lysine 45 (K45), resulting in proteasomal degradation and termination of autophagy.^[^ ^28^ ^]^ However, the sites at which AMBRA1 is ubiquitinated by other E3 ligases, such as CUL4, have not yet been determined. In this study, we identified two additional ubiquitination sites of AMBRA1 (K83+K86) that target AMBRA1 for protein degradation and are deubiquitinated by USP7 during the oxidative stress response. The discrepancy in these observations regarding the ubiquitination sites of AMBRA1 may be due to the use of different cell types (MEF vs 293T/IECs) and conditions (autophagy vs baseline/oxidative stress).

Intestinal oxidative stress is associated with the development of IBD. The activation of the transcription factor NRF2 is emerging as an attractive therapeutic strategy for attenuating oxidative stress and inflammation in IBD treatment.^[^ ^8^, ^9^, ^10^ ^]^ 5‐Aminosalicylic acid (5‐ASA), which is commonly used as an anti‐inflammatory treatment in patients with UC, can activate NRF2, thereby counteracting gut inflammation and oxidative stress.^[^ ^52^, ^53^ ^]^ NRF2 activators have also shown promising efficacy in DSS‐induced colitis mouse model.^[^ ^54^, ^55^ ^]^ However, the development of NRF2 activators is accompanied by various challenges including target specificity and safety considerations.^[^ ^56^ ^]^ Most NRF2 activators target KEAP1 or the protein‒protein interaction between KEAP1 and NRF2, which releases NRF2 from the CUL3‒KEAP1 E3 ubiquitin ligase complex and stabilizes NRF2.^[^ ^56^ ^]^ USP7 can stabilize KEAP1, and inhibition of USP7 can effectively activate NRF2 signaling, thereby alleviating neuroinflammation by promoting KEAP1 degradation.^[^ ^15^ ^]^ Here, we found that USP7 inhibition promoted AMBRA1 degradation, leading to an increased interaction between NRF2 and its deubiquitinase DUB3. Because inhibition of USP7 can activate NRF2 through both disruption of KEAP1‐dependent polyubiquitination and enhancement of DUB3‐dependent deubiquitination of NRF2, inhibition of USP7 could result in greater activation of NRF2 than that achieved when classical NRF2 activators targeting the KEAP1–NRF2 regulatory axis are used. Although NRF2 overactivation can also exacerbate colitis,^[^ ^57^ ^]^ we found that the USP7 inhibitor P5091 relieved oxidative stress and attenuated gut inflammation in a DSS‐induced colitis mouse model, suggesting the clinical translation of USP7 inhibitors for IBD treatment. In addition to 5‐ASA, biologics represented by anti‐TNF‐α are widely used in the treatment of IBD.^[^ ^58^ ^]^ However, biologics cannot induce sustained remission and mucosal healing in all patients,^[^ ^59^ ^]^ which may highlight the inadequacy of inhibiting inflammatory cytokines in the treatment of IBD. Our recent study indicated that AMBRA1 significantly inhibited the therapeutic effect of infliximab, the widely used monoclonal antibody against TNF‐α, and was highly expressed in patients with no clinical remission after anti‐TNF‐α therapy.^[^ ^23^ ^]^ Thus, targeting USP7/AMBRA1 complex could be a potential and effective strategy to inhibit intestinal oxidative stress and inflammation for IBD treatment.

**Figure 8:**
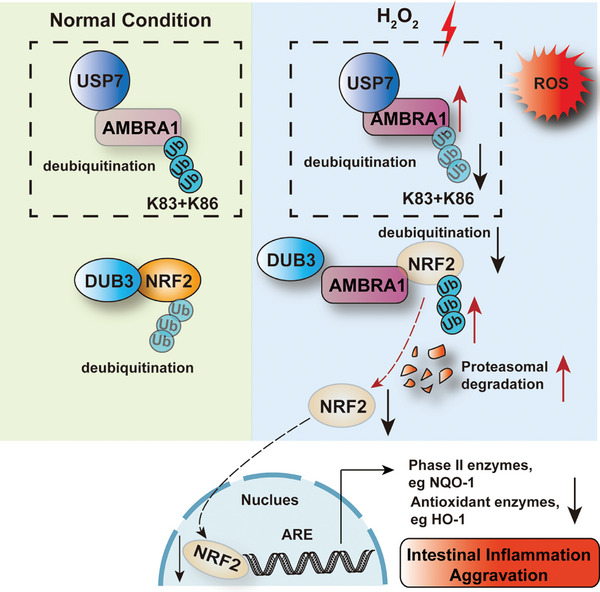
Schematic diagram illustrating the findings of this study. The deubiquitinase USP7 interacts with AMBRA1 and deubiquitinates it at K83/K86, thereby stabilizing the AMBRA1 protein. H_2_O_2_ stimulation promotes the interaction between AMBRA1 and USP7, thereby enhancing the deubiquitination and stabilization of AMBRA1 by USP7 in IECs under oxidative stress conditions. The F1 domain of AMBRA1 competitively inhibits the binding of DUB3 to the N‐terminal 1–350 domain of NRF2, leading to abrogated deubiquitination of NRF2 by DUB3, increased NRF2 degradation and subsequent decreased expression of HO‐1 and NQO‐1; these effects further promote intestinal oxidative stress and aggravate intestinal inflammation.

ROS has been reported as the inducer of autophagy.^[^ ^60^ ^]^ Both O_2_‐ and H_2_O_2_ are considered the primary ROS that induce autophagy after starvation, and antioxidant treatment prevents autophagy.^[^ ^61^, ^62^, ^63^ ^]^ In addition, oxygen‐glucose deprivation (OGD)‐induced generation of O_2_‐ or H_2_O_2_ promoted autophagy by inducing AMBRA1 expression in both human dental pulp stem cells (hDPSC) and human mesenchymal stem cells (hMSC).^[^ ^64^ ^]^ Thus, our finding that AMBRA1 inhibits NRF2 activity is consistent with the pro‐autophagic function of AMBRA1 during autophagy initiation. AMPK has been proposed to be activated upon H_2_O_2_ exposure through the S‐glutathionylation of reactive cysteines, which can activate ULK1 and subsequently phosphorylate and stabilize AMBRA1 to initiate autophagy.^[^ ^21^, ^65^, ^66^ ^]^ Additionally, H_2_O_2_ promoted the deubiquitination of AMBRA1 by USP7 in the nucleus, antagonizing DUB3‐mediated NRF2 deubiquitination and stabilization. We hypothesized that the suppressive effect of AMBRA1 on the NRF2‐induced antioxidant response might benefit autophagy initiation and clearance of damaged biomolecules and organelles under oxidative stress conditions. Taken together, our findings revealed that AMBRA1 plays a critical role in antagonizing DUB3‐mediated NRF2 deubiquitination, thereby promoting NRF2 degradation and aggravating oxidative stress (**Figure** **8**). They illustrated the novel possibility of targeting the USP7‐AMBRA1 axis to attenuate intestinal oxidative stress and inflammation in the treatment of IBD.

## 4 Experimental Section

### Cell Lines and Reagents

Human differentiated colorectal adenocarcinoma HT29 cells, human normal intestinal epithelial HIEC‐6 cells, and HEK293T cells were purchased from the American Type Culture Collection (Manassas, VA, USA). HT29 and HEK293T cells were cultured in DMEM supplemented with 10% fetal bovine serum (FBS), streptomycin and penicillin (Gibco, Grand Island, NY, USA) at 37 °C in 5% CO2. RPMI 1640 medium supplemented with 10% FBS, streptomycin, and penicillin (Gibco) was used for HIEC‐6 cell culture.

Hydrogen peroxide (H_2_O_2_) was purchased from Sinopharm Chemical Reagent Co. Ltd. The USP7 inhibitor P5091, proteasomal inhibitor MG132, and protein synthesis inhibitor cycloheximide were purchased from Selleck Chemicals and Sigma‒Aldrich (St. Louis, MO, USA). The antibodies used in this study were as follows: anti‐AMBRA1 (13762‐1‐AP, Proteintech (Rosemont, IL, USA), 1:1000 for WB, 1:500 for IP and 1:100 for immunohistochemistry [IHC]), anti‐NRF2 (16396‐1‐AP, Proteintech, 1:1000 for WB, 1:100 for IHC and IF), anti‐HO‐1 (10701‐1‐AP, Proteintech, 1:1000 for WB and 1:100 for IHC), anti‐NQO‐1 (11451‐1‐AP, Proteintech, 1:1000 for WB and 1:100 for IHC), anti‐DUB3 (26143‐1‐AP, Proteintech, 1:1000 for WB), anti‐USP7 (66514‐1‐Ig, Proteintech, 1:1000 for WB), anti‐HA (3724, Cell Signaling Technology [Beverly, MA, USA], 1:1000 for WB), anti‐FLAG (14 793, Cell Signaling Technology, 1:1000 for WB), anti‐MYC (2276, Cell Signaling Technology, 1:1000 for WB), anti‐β‐actin (A2228, Sigma‒Aldrich, 1:10000 for WB) and anti‐Lamin A (10298‐1‐AP, Sigma‒Aldrich, 1:10000 for WB). Normal rabbit IgG (2729; Cell Signaling Technology) was used.

The pRK7‐FLAG‐AMBRA1, pcDNA‐HA‐AMBRA1, pcDNA‐MYC‐AMBRA1, pRK7‐FLAG‐KEAP1, pCDNA‐GFP‐NRF2, pcDNA‐HA‐NRF2, pRK7‐FLAG‐DUB3, pcDNA‐HA‐DUB3, pRK7‐FLAG‐USP7, pcDNA‐HA‐USP7, and pcDNA‐MYC‐USP7 vectors were constructed using the ClonExpress I One Step Cloning Kit (Vazyme, Nanjing, China). HA‐Ub, HA‐Ub‐K48R, and HA‐Ub‐K63R plasmids were kindly provided by Dr. Wei Yu of Fudan University. Expression plasmids for mutant AMBRA1, KEAP1, and USP7 were generated using a KOD mutagenesis kit (Toyobo, Osaka, Japan) according to the manufacturer's instructions.

### Immunoblotting, Immunoprecipitation and Ubiquitination Assays

For direct western blot analysis, cells were lysed in NP‐40 lysis buffer (1% NP40 NaF, 50 mM Tris‐HCl (pH 7.5), 150 mM NaCl, 1 mM PMSF, 25 mM NaF, and 1 mM Na_3_VO_4_) supplemented with a protease inhibitor cocktail (Roche). For regular immunoprecipitation, cells were lysed in 0.3% NP40 lysis buffer and then subjected to immunoprecipitation using anti‐FLAG/HA/GFP magnetic beads (Bimake) for 3 h at 4 °C. For immunoprecipitation for ubiquitination detection, the cells were pretreated with MG132 for 4 h before cell lysis. The cells were then lysed in 100 µL of 1% SDS lysis buffer containing 1 mM PMSF. After immediate boiling at 95 °C for 5 min, the cell lysates were diluted by addition of 900 µL of 0.3% NP40 complete lysis buffer for subsequent immunoprecipitation. For endogenous immunoprecipitation, the cell lysates were incubated with the indicated primary antibodies or control IgG conjugated to protein A/G agarose overnight at 4 °C. The precipitated proteins were boiled and eluted from the beads at 95 °C for 10 min for immunoblotting. To enhance the detection of NRF2 basal protein level, 60–80 µg total protein was used for western‐blot analysis of NRF2 in HEK293T cells.

### Quantitative Real‐Time PCR

Total RNA was extracted from tissues or cells using the TRIzol reagent (Takara, Japan). Reverse transcription was performed using a PrimeScript RT Master Mix Kit (Takara, Japan). SYBR Premix ExTaq (Yeasen, Shanghai, China) and an Applied Biosystems 7500 Fast Real‐Time PCR system were used for qPCR analysis. The relative expression level of mRNA was evaluated via the 2^−ΔΔCt^ method; expression levels were normalized to the level of β‐actin expression. All experiments were performed in triplicates. Detailed sequences of the qPCR primers used in this study were presented in Table S2 (Supporting Information).

### Human Tissue Samples and Immunohistochemistry

Inflamed and paired noninflamed colon tissues were obtained from patients with IBD who underwent colectomy at the Department of Colorectal Surgery, Xinhua Hospital, Shanghai Jiaotong University School of Medicine. This study was approved by the Ethics Committee of Xinhua Hospital (No. XHEC‐NSFC‐2022‐113). Informed consent was obtained from all participants. Immunohistochemical staining was performed according to a standard protocol, using the heat‐induced epitope retrieval method. AMBRA1, NRF2, USP7, HO‐1, and NQO‐1 expression levels in intestinal epithelial cells were semiquantitatively evaluated by two independent pathologists. The final scores were calculated by multiplying the proportion score by the intensity score.^[^ ^23^ ^]^

### Immunofluorescence and Proximity Ligation Assays

For immunofluorescence (IF) staining of patient samples and mouse colon tissues, an SABC‐Cy3 kit was used, according to the manufacturer's instructions. For immunofluorescence staining, cells grown on coverslips were fixed with 4% paraformaldehyde (PFA) and permeabilized with 0.1% Triton X‐100. Cells were blocked with 5% bovine serum albumin and incubated overnight with the indicated primary antibodies. Alexa Fluor 488‐conjugated donkey anti‐rabbit IgG (Life Technologies, Grand Island, NY, USA) was used as the secondary antibody. The slides were counterstained with DAPI and analyzed by fluorescence microscopy. PLA was performed using a Duolink In Situ Detection Kit (Sigma, #DUO92008) according to the manufacturer's instructions. The cells plated on glass coverslips were fixed, permeabilized, blocked, and incubated with primary antibodies. The cells were then hybridized to PLA probes. After the ligation and amplification of the PLA signals, the PLA puncta were photographed and counted for statistical analysis.

### Mice and Mouse Model of DSS‐Induced Colitis

*Ambra1^flox/flox^* mice and *Villin‐Cre* mice with a C57BL/6 background were purchased from the Shanghai Model Organisms Center (Stock No: NM‐CKO‐200059) and Jackson Laboratory (Stock No: 0 04586), respectively, and crossed to generate *Villin‐Ambra1^flox/flox^* mice. DSS (3%) was dissolved in drinking water to induce colitis in the mice. Eight‐week‐old male mice (20–24 g) were randomly assigned to control or DSS groups, and their body weights, activity levels, and changes in stool characteristics were monitored. DAI evaluation and histopathological scoring were performed as described previously. Briefly, the DAI includes evaluations of body weight, stool characteristics and the degree of rectal bleeding, which were scored on a scale of 0–4.^[^ ^67^ ^]^ Each animal's total score was then divided by 3 to obtain a DAI score. Histopathological scores were evaluated based on neutrophil infiltration, absence of crypts, cross‐sectional involvement, and erosion or ulceration in hematoxylin‐eosin‐stained slides of mouse intestines.^[^ ^68^ ^]^ The colorectum and spleen of the animals were dissected to compare their length and weight. Additionally, colorectal tissues from the indicated groups were fixed with 4% PFA and used to examine oxidative stress markers. To evaluate the effect of the specific USP7 inhibitor, P5091, on DSS‐induced colitis, mice were injected intraperitoneally three times during the induction process with P5091 (20 mg k^−1^g) prepared in a solution (20% DMSO, 40% PEG‐300, or 40% PBS). Littermate and male mice were used for the construction of DSS‐induced colitis model in this study. All mice were randomly assigned to the indicated groups and sacrificed for subsequent assessment after 7 days of DSS treatment. All animal experimental procedures were approved by the Laboratory Animal Care and Welfare Committee of Xinhua Hospital affiliated to Shanghai Jiaotong University School of Medicine (No. XHEC‐F‐2023‐039).

### Statistical Analysis

SPSS version 19.0 (IBM 2010, Chicago, IL, USA) and GraphPad Prism 8.0 (San Diego, CA, USA) were used for statistical analysis. The quantitative data were presented as the means ± standard deviations (SDs). A two‐tailed unpaired Student's *t*‐test was used to analyze the significance of two‐group comparisons. One‐way ANOVA was used to assess the statistical significance of experiments with >2 independent groups. For DAI assessments, a two‐way ANOVA was performed to assess statistical significance. Spearman's correlation analysis was used to assess the correlation between AMBRA1 expression and the expression of NRF2, HO‐1, and NQO‐1. Sample size for each statistical analysis was presented in corresponding figure legends. All statistical tests were two‐sided, and *p* values <0.05 were considered statistically significant.

## Conflict of Interest

The authors declare no conflict of interest.

## Author Contributions

W.X., Z.H., and Y.W. contributed equally to this work. P.D., C.‐Y.L, and Y.G. designed the research. W.X., Z.H., and Y.W. performed experiments and/or analyzed data. W.X. and C.‐Y.L. wrote the manuscript. W.T. assisted some analysis and collected the specimens from patients. W.G., Y.C., and Z.W. reviewed and revised the manuscript. All authors approved the final version.

## Supporting information

Supporting Information

Table S1

## Data Availability

The raw data supporting the conclusions of this article will be made available by the authors, without undue reservation.
