# Supplementary material for: Redox‐Induced Stabilization of AMBRA1 by USP7 Promotes Intestinal Oxidative Stress and Colitis Through Antagonizing DUB3‐Mediated NRF2 Deubiquitination
Source: Adv Sci (Weinh). 2025 Jan 31;12(12):2411320. doi: 10.1002/advs.202411320 (PMC11948009; doi:10.1002/advs.202411320)
Supplement: Supplementary file 1 — Supporting Information [file ADVS-12-2411320-s002.docx]

**Supplementary Figures**

**
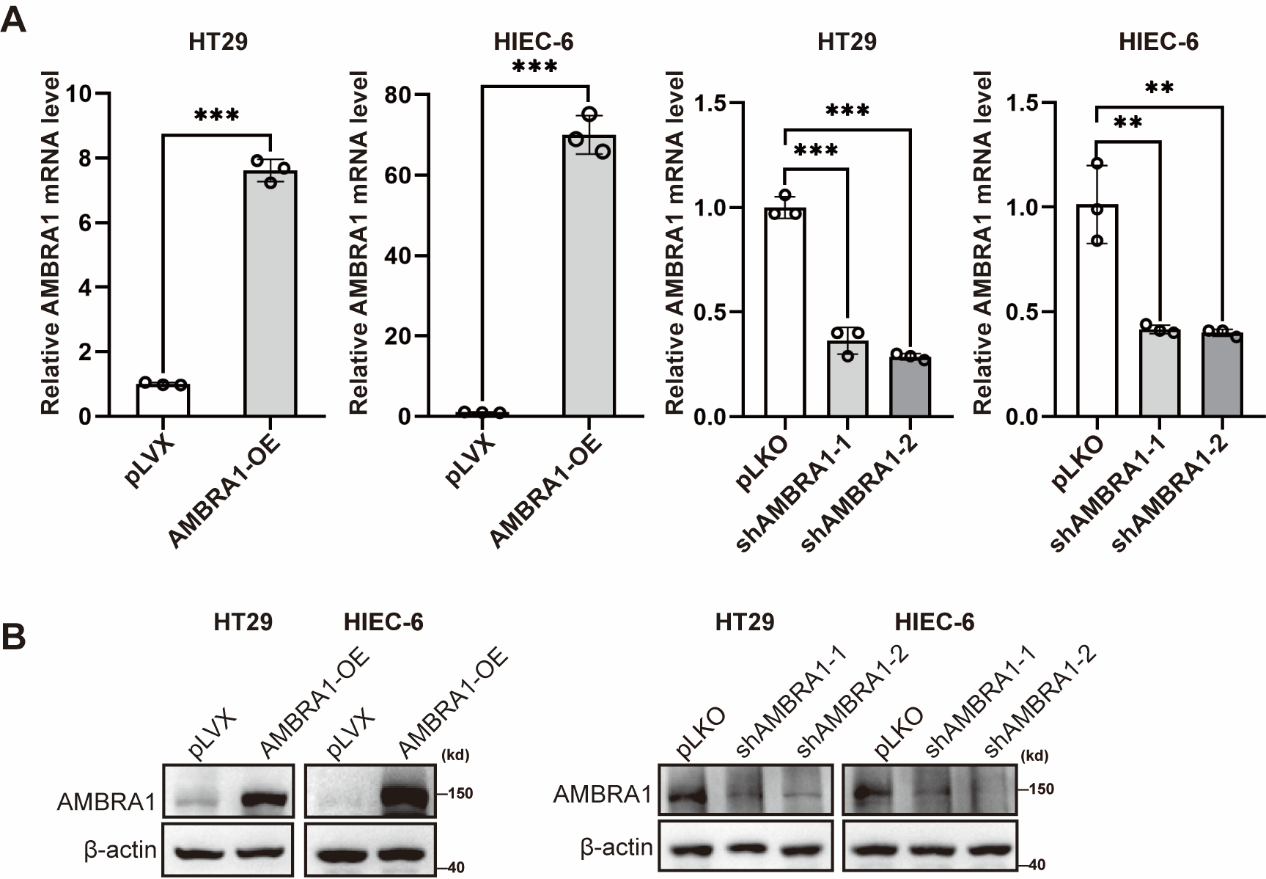
**

**Figure S1.** **Confirmation of AMBRA1-knockdown and overexpression in HT29 and HIEC-6 cells.** (A-B) The knockdown efficiency and overexpression of AMBRA1 in HT29 and HIEC-6 cells were examined via qRT‒PCR (A) and western blot analysis (B). The data are presented as the means ± SDs. n=3 (A) biologically independent samples per group. These data (A) are representative of 2 independent experiments. Two-tailed, unpaired Student’s t test and one-way ANOVA were performed to assess statistical significance. ** P < 0.01, *** P < 0.001.


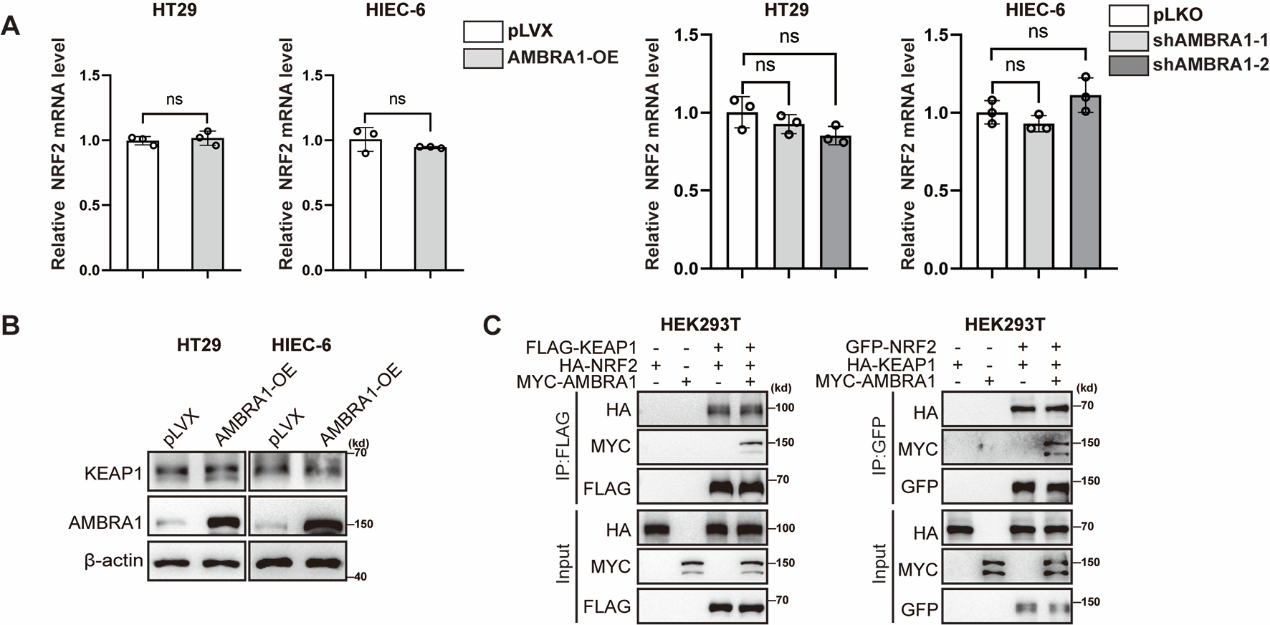


**Figure S2. AMBRA1 has no effect on NRF2 transcription and KEAP1 expression.** (A) qRT‒PCR analysis of NRF2 mRNA levels in AMBRA1-overexpressing, AMBRA1-knockdown and control HT29 and HIEC-6 cells. (B) Western blot analysis of KEAP1 expression in AMBRA1-overexpressing HT29 and HIEC-6 cells. (C) Exogenous co-IP of FLAG-KEAP1 and HA-NRF2 with or without co-expression of MYC-AMBRA1 and exogenous co-IP of GFP-NRF2 and HA-KEAP1 with or without co-expression of MYC-AMBRA1 in HEK293T cells. These data (A) are representative of 2 independent experiments. Two-tailed, unpaired Student’s t test and one-way ANOVA were performed to assess statistical significance. ** P < 0.01, *** P < 0.001.


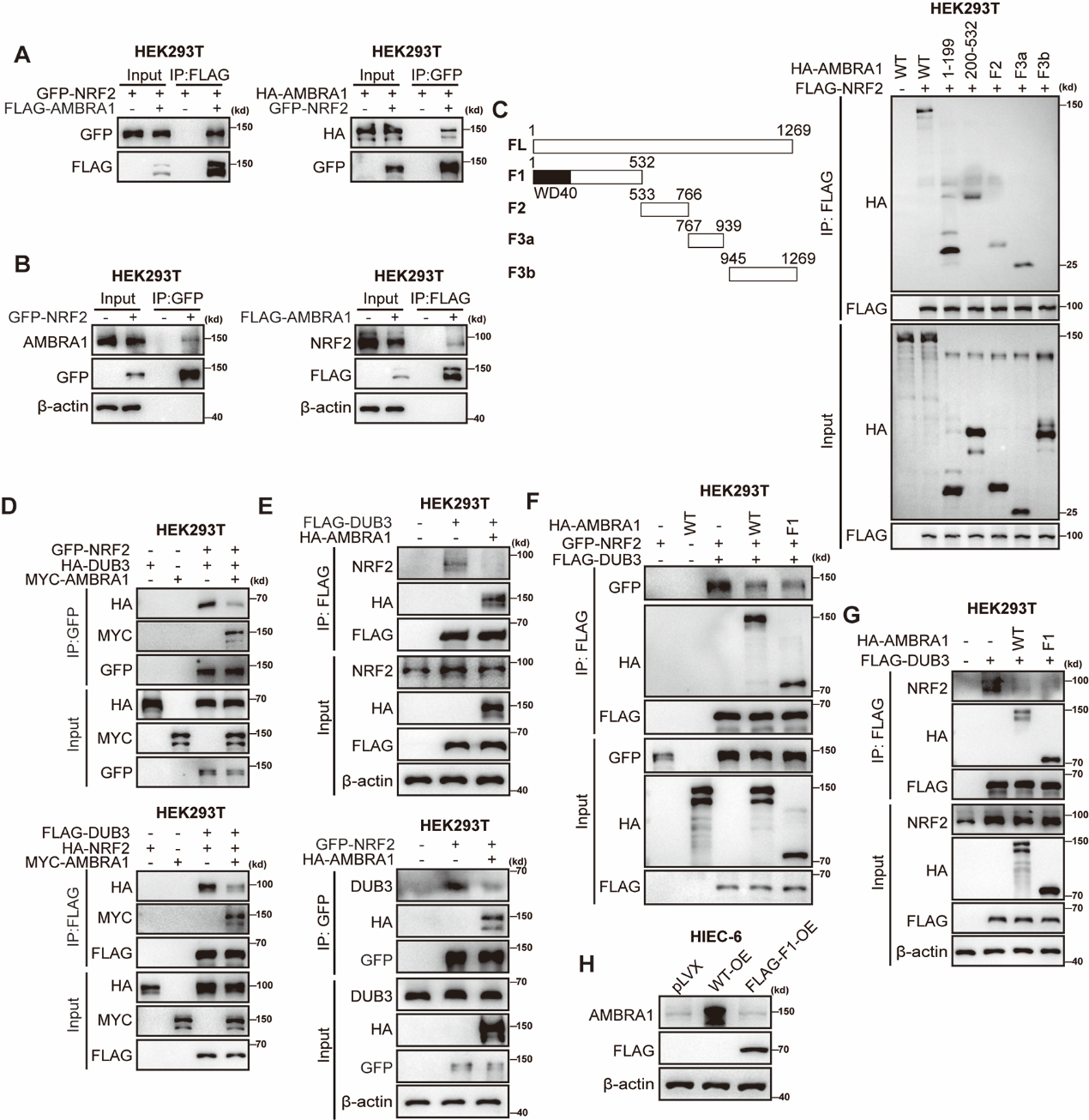


**Figure S3. AMBRA1 competes with DUB3 for interaction with NRF2.** (A) Exogenous co-IP of FLAG-AMBRA1 and GFP-NRF2 or HA-AMBRA1 and GFP-NRF2 in HEK293T cells. (B) Semi-endogenous co-IP of exogenous GFP-NRF2, FLAG-AMBRA1 and endogenous AMBRA1 and NRF2 in HEK293T cells. (C) Exogenous co-IP of FLAG-NRF2 and full-length/truncated HA-AMBRA1 in HEK293T cells. (D) Exogenous co-IP of GFP-NRF2 and HA-DUB3 with or without co-expression of MYC-AMBRA1 and exogenous co-IP of FLAG-DUB3 and HA-NRF2 with or without co-expression of MYC-AMBRA1 in HEK293T cells. (E) Semi-endogenous co-IP of exogenous FLAG-DUB3 or GFP-NRF2 and endogenous NRF2 or DUB3 with or without co-expression of HA-AMBRA1 in HEK293T cells. (F) Exogenous co-IP of FLAG-DUB3 and GFP-NRF2 in HEK293T cells overexpressing full-length HA-AMBRA1 or the F1 fragment of HA-AMBRA1. (G) Semi-endogenous co-IP of exogenous FLAG-DUB3 and endogenous NRF2 in HEK293T cells overexpressing full-length HA-AMBRA1 or the F1 fragment of HA-AMBRA1. (H) HIEC-6 cells stably overexpressing AMBRA1 or its F1 fragment were established and confirmed via western blotting.

**
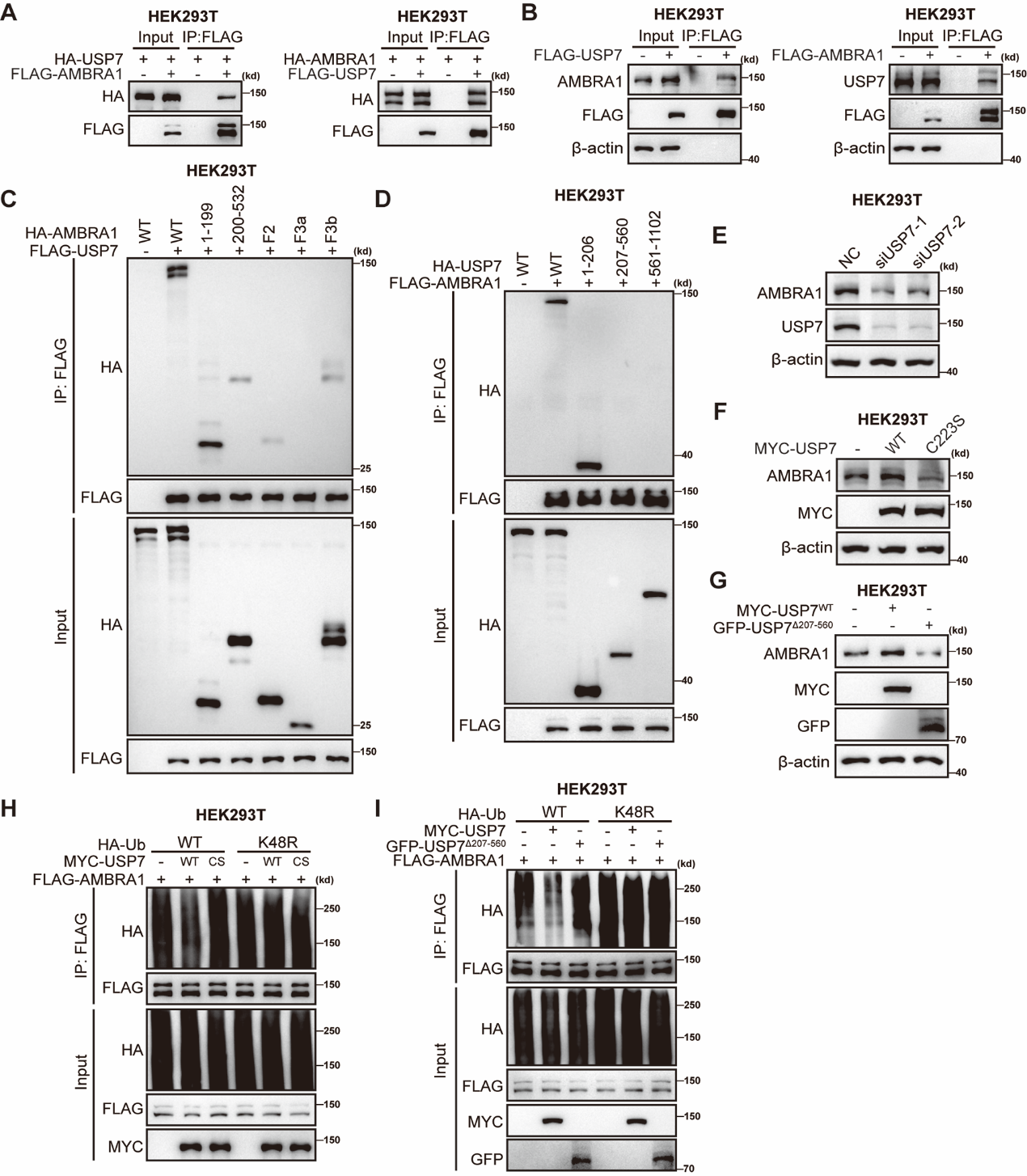
**

**Figure S4. USP7 interacts with and stabilizes the AMBRA1 protein.** (A) Exogenous co-IP of FLAG-AMBRA1 and HA-USP7, FLAG-USP7 and HA-AMBRA1 in HEK293T cells. (B) Semi-endogenous co-IP of exogenous FLAG-USP7, FLAG-AMBRA1 and endogenous AMBRA1 and USP7 in HEK293T cells. (C) Exogenous co-IP of FLAG-USP7 and full-length/truncated HA-AMBRA1 in HEK293T cells. (D) Exogenous co-IP of FLAG-AMBRA1 and full-length/truncated HA-USP7 in HEK293T cells. (E) The protein level of AMBRA1 was examined via western blotting in HEK293T cells transfected with control and siRNA targeting USP7. (F-G) HEK293T cells were transfected with the indicated control, MYC-USP^WT^, MYC-USP7^C223S^, and GFP-USP7^Δ207-506^ plasmids. AMBRA1 expression was determined via western blotting. (H-I) HEK293T cells were transfected with HA-Ub, HA-Ub-K48R, MYC-USP^WT^, MYC-USP7^C223S^ or GFP-USP7^Δ207-506^ and FLAG-AMBRA1. FLAG-AMBRA1 was immunoprecipitated, and its ubiquitination level was measured via western blotting.

**
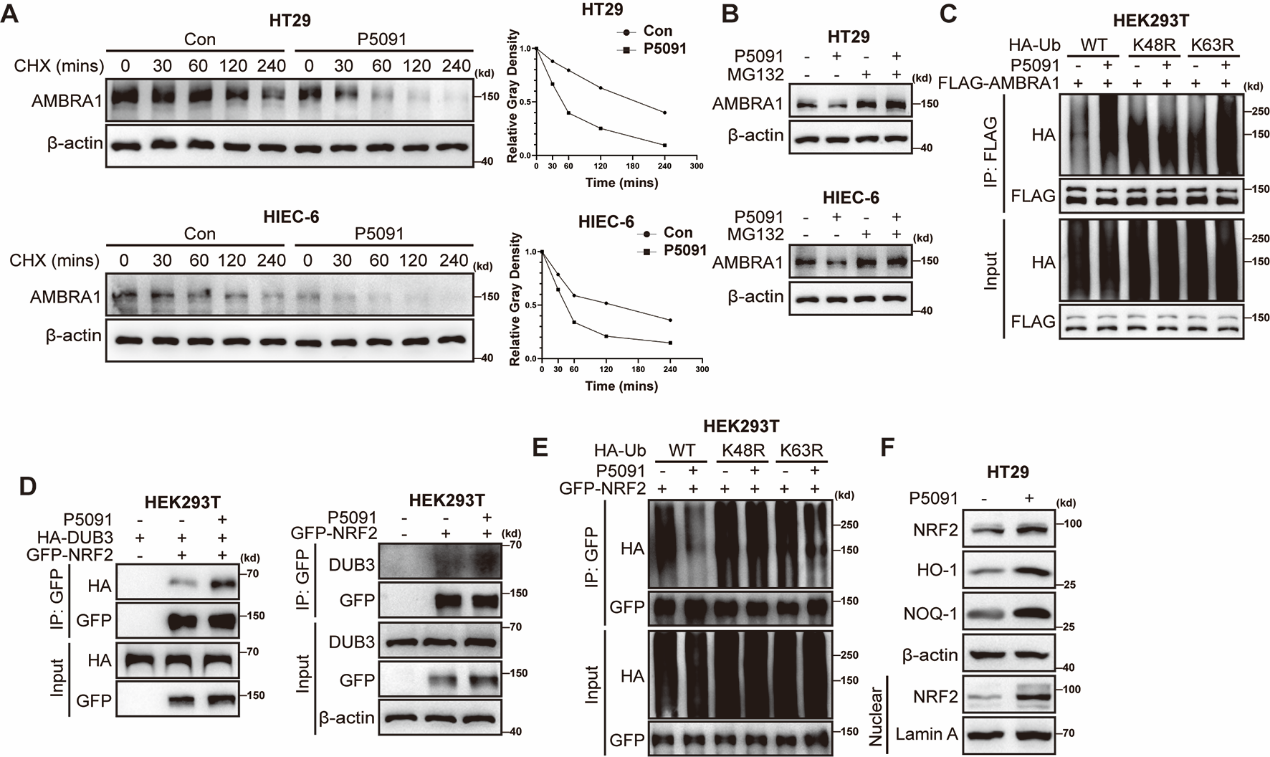
**

**Figure S5. The USP7 inhibitor P5091 decreases AMBRA1 expression and thereby stabilizes the NRF2 protein.** (A) Analysis of the half-life of AMBRA1 protein in HT29 and HIEC-6 cells with or without P5091 (10 μM) treatment. The cells were treated with CHX (75 μg/ml) for the indicated times before western blot analysis of the AMBRA1 protein. (B) Western blot analysis of AMBRA1 expression in HT29 and HIEC-6 cells treated with P5091 (10 μM) alone or in combination with MG132 (10 μM). (C) HEK293T cells were transfected with the HA-Ub, HA-Ub-K48R, HA-Ub-K63R, FLAG-AMBRA1 plasmid and treated with or without P5091(10 μM). The cell lysates were used for immunoprecipitation with an anti-FLAG antibody for subsequent analysis of AMBRA1 ubiquitination via western blotting. (D) Exogenous co-IP of GFP-NRF2 and HA-DUB3 and semiendogenous co-IP of exogenous GFP-NRF2 and endogenous DUB3 in HEK293T cells with or without P5091 (10 μM) treatment. (E) HEK293T cells were transfected with the HA-Ub, HA-Ub-K48R, HA-Ub-K63R, GFP-NRF2 plasmid and treated with or without P5091 (10 μM). The cell lysates were used for immunoprecipitation with an anti-GFP antibody for subsequent analysis of NRF2 ubiquitination via western blotting. (F) Western blot analysis of the total protein levels of NRF2, HO-1, and NQO-1 and the nuclear expression level of NRF2 in HT29 cells with or without P5091 (10 μM) treatment.


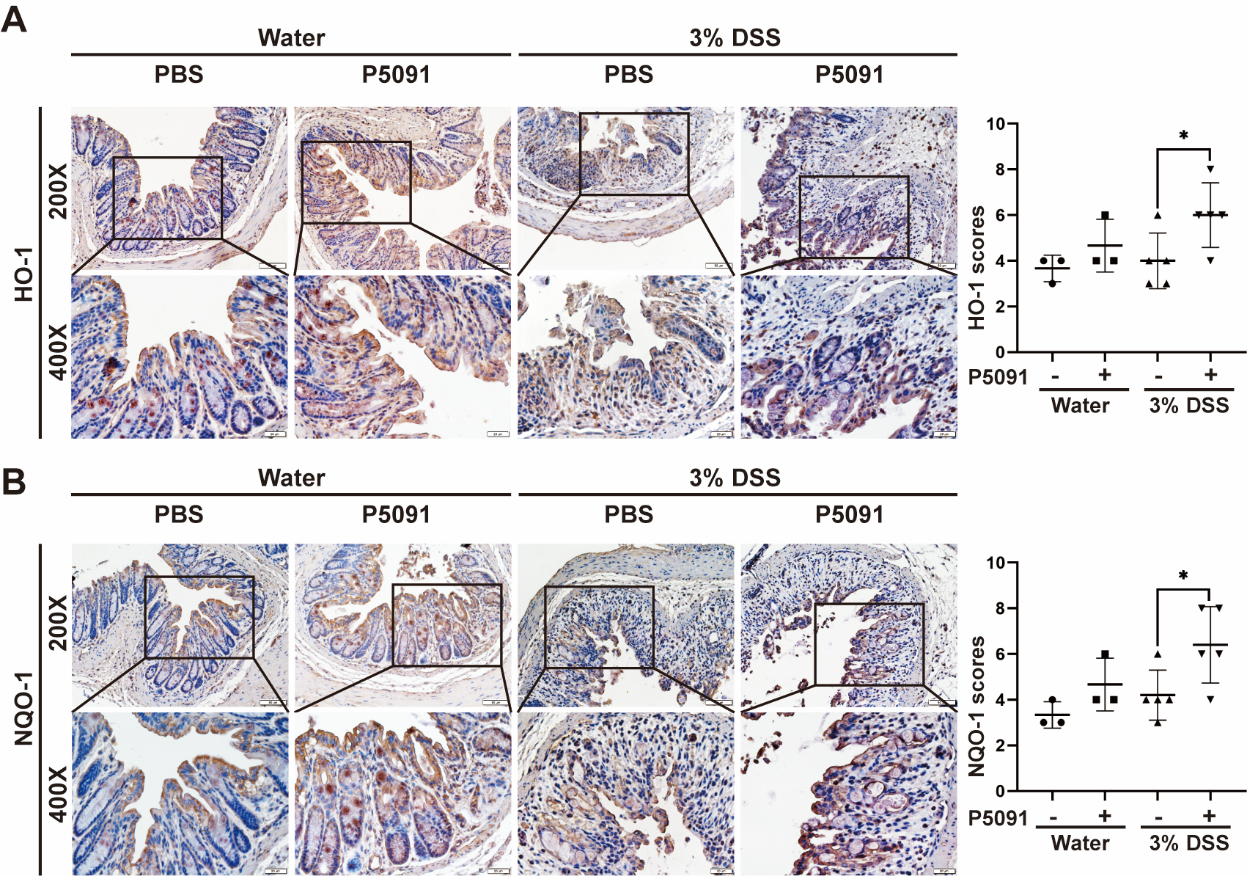


**Figure S6.** **USP7 inhibitor P5091 diminishes oxidative stress and attenuates DSS-induced colitis *in* *vivo.*** The expression of HO-1 (A) and NQO-1 (B) were examined in control and P5091-treated mice via immunohistochemistry. The data are presented as the means ± SDs. n=3 biologically independent samples for control group, n=5 biologically independent samples for DSS group The Mann‒Whitney’s U test (A, B) were performed to assess statistical significance. * *P* < 0.05.


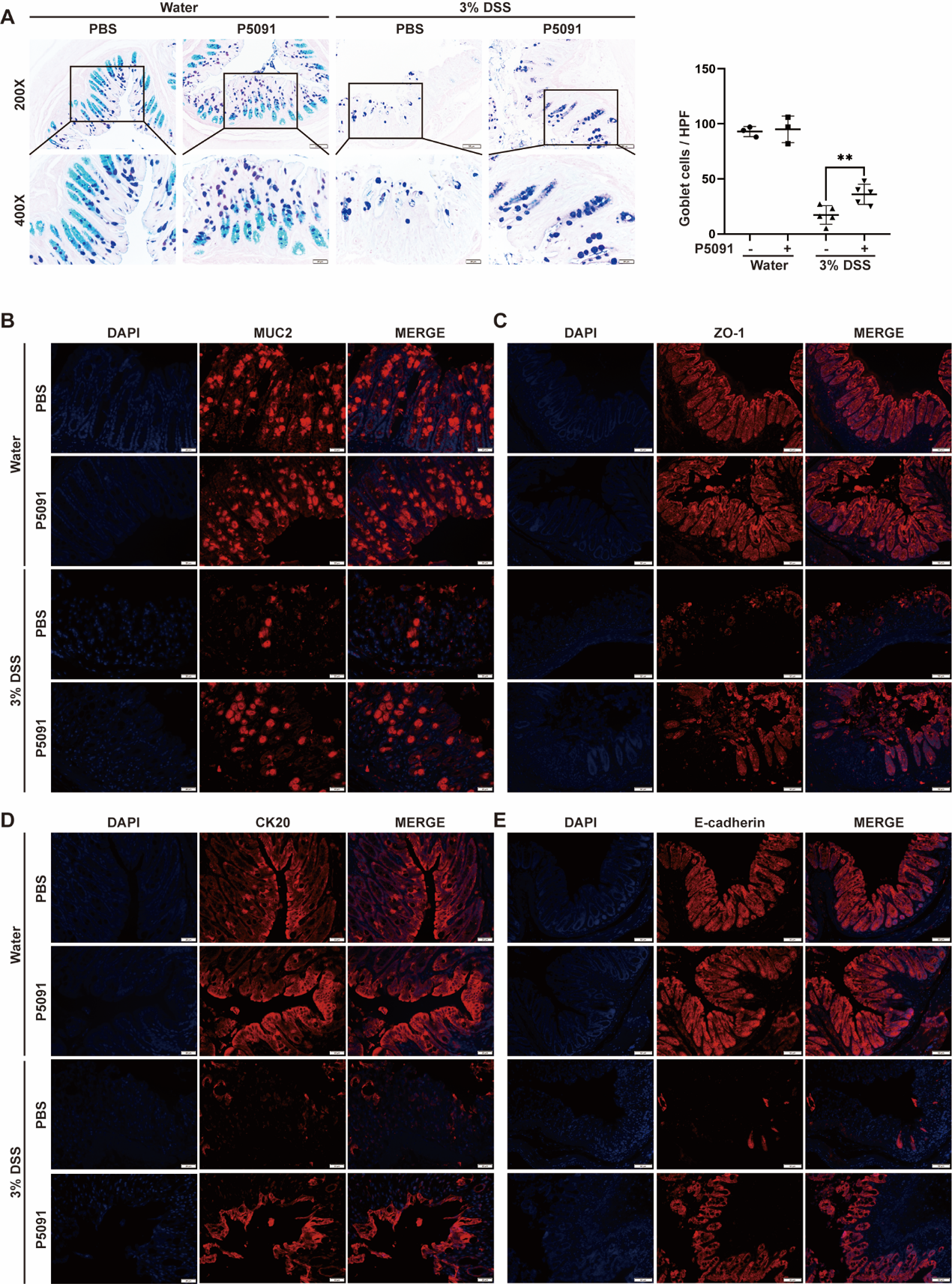


**Figure S7. P5091 ameliorates DSS-induced intestinal barrier damage *in vivo*.** (A) Alcian blue/periodic acid-Schiff staining of goblet cells in colon tissues from control and P5091-treated mice (magnification: upper panels, 200×; lower panels, 400×). (B-E) Representative immunofluorescence images showing MUC2 (B), ZO-1 (C), CK20 (D) and E-cadherin (E) in control and P5091-treated groups (magnification: upper panels, 200×; lower panels, 400×). The data are presented as the means ± SDs. n=3 biologically independent samples for control group, n=5 biologically independent samples for DSS group (A). One-way ANOVA (A) was performed to assess statistical significance. ** *P* < 0.01.

| **Table S2: The primers, shRNA and siRNA sequence used in this study.** | | |
| --- | --- | --- |
| **Primers** | **SEQUCENCE** |  |
| *AMBRA1 (human)* | Forward: AACCCTCCACTGCGAGTTGA | |
|  | Reverse: TCTACCTGTTCCGTGGTTCTCC | |
| *NRF2 (human)* | Forward: TCAGCGACGGAAAGAGTATGA | |
|  | Reverse: CCACTGGTTTCTGACTGGATGT | |
| *β-actin (human)* | Forward: CATGTACGTTGCTATCCAGGC | |
|  | Reverse: CTCCTTAATGTCACGCACGAT | |
| AMBRA1-sh1 | 5′- GGCCTATGGTACTAACAAA -3′ | |
| AMBRA1-sh2 | 5′- GAGATTATCTCCTGCTGCATA -3′ | |
| siAMBRA1-1 | AGAACTGCAAGATCTACAA | |
| siAMBRA1-2 | GGCCTATGGTACTAACAAA | |
| siKEAP1-1 | CCUCAAUCGUCUCCUUUAUTT | |
| siKEAP1-2 | GGCGAAUGAUCACAGCAAUTT | |
| siDUB3-1 | CAGCUUGCUCCCAGGAAGATT | |
| siDUB3-2 | UGCCAAGGUCACUGCCUGUTT | |
